# *GNG5* is a novel oncogene associated with cell migration, proliferation, and poor prognosis in glioma

**DOI:** 10.1186/s12935-021-01935-7

**Published:** 2021-06-07

**Authors:** Wang Zhang, Zhendong Liu, Binchao Liu, Miaomiao Jiang, Shi Yan, Xian Han, Hong Shen, Meng Na, Yanbiao Wang, Zhishuai Ren, Binfeng Liu, Zhenfeng Jiang, Yanzheng Gao, Zhiguo Lin

**Affiliations:** 1 Department of Neurosurgery, The First Affiliate Hospital of Harbin Medical University, 23 Youzheng Street, Nangang District, Harbin, 150001 China; 2 Department of Orthopaedics, Department of Microbiome Laboratory, Henan Provincial People’s Hospital, People’s Hospital of Zhengzhou University, School of Clinical Medicine, Henan University, No. 7, Weiwu Road, Jinshui District, Zhengzhou, 450003 Henan China; 3 Department of Neurosurgery of Xing, Tai People’s Hospital, Xing Tai, China; 4 Department of the Pathology, The First Affiliate Hospital of Harbin Medical University, Harbin, China

**Keywords:** Glioma, Oncogene, Biomarker, GNG5

## Abstract

**Background:**

Although many biomarkers have been reported for detecting glioma, the prognosis for the disease remains poor, and therefore, new biomarkers need to be identified. *GNG5*, which is part of the G-protein family, has been associated with different malignant tumors, though the role of *GNG5* in glioma has not been studied. Therefore, we aimed to identify the relationship between *GNG5* and glioma prognosis and identify a new biomarker for the diagnosis and treatment of gliomas.

**Methods:**

We used data on more than a thousand gliomas from multiple databases and clinical data to determine the expression of *GNG5* in glioma. Based on clinical data and CGGA database, we identified the correlation between *GNG5* and multiple molecular and clinical features and prognosis using various analytical methods. Co-expression analysis and GSEA were performed to detect *GNG5*-related genes in glioma and possible signaling pathways involved. ESTIMATE, ssGSEA, and TIMER were used to detect the relationship between *GNG5* and the immune microenvironment. Functional experiments were performed to explore the function of *GNG5* in glioma cells.

**Results:**

*GNG5* is highly expressed in gliomas, and its expression level is positively correlated with pathological grade, histological type, age, and tumor recurrence and negatively correlated with isocitrate dehydrogenase mutation, 1p/19 co-deletion, and chemotherapy. Moreover, *GNG5* as an independent risk factor was negatively correlated with the overall survival time. GSEA revealed the potential signaling pathways involved in *GNG5* function in gliomas, including cell adhesion molecules signaling pathway. The ssGSEA, ESTIMATE, and TIMER based analysis indicated a correlation between *GNG5* expression and various immune cells in glioma. In vivo and in vitro experiments showed that *GNG5* could participate in glioma cell proliferation and migration.

**Conclusions:**

Based on the large data platform and the use of different databases to corroborate results obtained using various datasets, as well as in vitro and in vivo experiments, our study reveals for the first time that *GNG5*, as an oncogene, is overexpressed in gliomas and can inhibit the proliferation and migration of glioma cells and lead to poor prognosis of patients. Thus, *GNG5* is a potential novel biomarker for the clinical diagnosis and treatment of gliomas.

**Supplementary Information:**

The online version contains supplementary material available at 10.1186/s12935-021-01935-7.

## Background

Glioma is the most common malignant tumor of the central nervous system (CNS), showing high recurrence and a high mortality rate [1]. Although extensive research has been performed on the etiology, diagnosis, and treatment of gliomas, the prognosis is still unsatisfactory despite active comprehensive treatment being adopted for glioma patients, including maximum surgical resection, postoperative radiotherapy, and chemotherapy [2]. To improve the diagnostic accuracy of glioma and predict the prognosis of glioma patients more accurately, The World Health Organization (WHO) in 2016 defined the subclassification of glioma using molecular parameters including the co-deletion of chromosomal 1p and 19q(chr1p/19q), and the mutational status of isocitrate dehydrogenase (IDH) [1]. However, as malignant tumors are highly heterogeneous, using these widely used biological markers is not sufficient for the precise diagnosis and treatment of glioma. Therefore, there is an urgent need for novel molecular markers with high specificity and sensitivity to improve the molecular diagnosis and treatment of glioma.

G-proteins are a family of proteins that participate in multiple cellular functions such as cell division, differentiation, and metastasis during embryonic development through direct interaction with G protein-coupled receptors [3, 4]. The formation of gliomas is related to embryonic development; for example, the epithelial-mesenchymal transition (EMT) is a process vital for embryonic development, and it has been shown to regulate the progression and invasion of glioma [5, 6]. Multiple studies have confirmed the significance of G-protein family members in the pathological progress of cancer. For example, *GNG7* is an epigenetic silencing gene involved in the malignant progression of renal clear cell carcinoma and esophageal cancer [7, 8]; *GNG4* function shows a high degree of correlation with liver cancer and colorectal cancer and is used in their diagnosis and prognosis [9, 10]; and *GNG11* promotes the adhesion, migration, and invasion of gastric cancer cells [11]. Thus, the G-protein family is associated with tumor progression and may be used as potential biomarkers for the diagnosis and treatment of tumors.

We focused on the *GNG5* protein of the G-protein family, and few reports describe the role of *GNG5* in tumors. *GNG5*, as a gamma subunit of G-protein, localized to the human chromosome 1p22, and is composed of 3 exons and 2 introns, and regulates the occurrence and development of many diseases [12]. Additionally, it acts as a regulator of islet function regulating the secretion of insulin, and is also used as a marker of melanosis coli to regulate cellular apoptosis [13, 14]. *GNG5* has been shown to play an important role in various cancers such as endometrial carcinoma [15] where it is highly expressed, and in invasive ductal carcinoma of the breast [16] where it regulates the secretion of E-cadherin through the Wnt signaling pathway. However, the role of *GNG5* in contributing to the pathological mechanism in glioma has not been reported.

We focused on exploring the expression pattern of *GNG5* in glioma from gene level to protein level, examining the underlying relationship between *GNG5* and the clinical and molecular characteristics of glioma in patients, and revealing the potential biological function of *GNG5* in the pathological progression of glioma. The results obtained could enhance our understanding of the complex molecular mechanisms of glioma and provide a promising novel target for combating the disease.

## Methods

### Data collection

A comparison of the transcript levels of *GNG5* in different tumors was performed using Gene Expression Profiling Interactive Analysis (GEPIA, http://gepia.cancer-pku.cn). To identify differences in expression for *GNG5* between gliomas and the control group, RNAseq data from 698 gliomas and 5 normal brain tissues were downloaded from The Cancer Genome Atlas (TCGA, https://portal.gdc.cancer.gov/), and microarray data of the GSE131273 dataset from the Gene Expression Omnibus (GEO, https://www.ncbi.nlm.nih.gov/geo/) database. RNA sequencing data from 1018 gliomas and their corresponding clinical information was downloaded from the Chinese Glioma Genome Atlas (CGGA, http://www.cgga.org.cn/help.jsp) database. The relationship between the expression level of *GNG5* and clinicopathological features was further analyzed using the data obtained from CGGA. The patients were divided into older and younger groups according to the median age of the patients, and high and low expression groups according to the average expression level of *GNG5* in the samples. Additionally, we obtained the overall survival (OS) information for glioma patients from the GSE53733 dataset, including 23 patients who showed long-term survival (> 36 months), 16 patients that showed short-term survival (< 12 months), and 31 patients who displayed intermediate survival. We analyzed the difference in expression of *GNG5* among the three groups of patients with different survival phenotypes. The Human Protein Atlas (HPA, https://www.proteinatlas.org/) was used to validate GNG5 expression at the protein level.

### Gene set enrichment analysis (GSEA) and immune correlation analysis

GSEA is a tool developed by research teams at MIT and Harvard University's Broad Institute to analyze genome-wide expression profiling from microarray data [17], and we used it for analyzing *GNG5* related pathways and molecular mechanisms in glioma. We used 1000 gene set permutations for each analysis using the expression level of *GNG5* as a reference phenotype tag. The resulting enriched pathways were analyzed based on nominal (NOM) *P*-values and normalized enrichment scores (NES). We examined the relationship between the expression of *GNG5* in glioma tissues and the immune microenvironment using the single-sample gene set enrichment analysis (ssGSEA) to calculate the enrichment of 29 immune cell geneset signatures in each glioma sample based on data downloaded from the CGGA database [18–20]. Further, a cluster analysis of glioma samples was performed based on the quantitative results obtained from the ssGSEA analysis. The ESTIMATE method (estimate R package) was used to evaluate the immune scores, tumor purity, and the stromal score for each glioma sample [21]. Tumor IMmune Estimation Resource (TIMER, https://cistrome.shinyapps.io/timer/) is an ideal public database for studying the infiltration abundance of tumor-infiltrating immune cells. The database pre-evaluated the immune infiltration levels of six immune infiltrating cells (including B cells, CD4 + T cells, CD8 + T cells, neutrophils, macrophages and dendritic cells) in 10897 cancer samples by complex statistical methods. We examined the relationship between the expression of GNG5 and the infiltration abundance of the above six immune cells in glioma using TIMER [22].

### Patients and tissue preparation

Forty primary glioma samples and five non-glioma samples were selected from the Department of Neurosurgery of the First Affiliated Hospital of Harbin Medical University. The tissues were cut into tissue blocks (5 mm thick). One block was fixed overnight in 4% paraformaldehyde, dehydrated, and embedded in paraffin, and the others were frozen in liquid nitrogen.

### Cell lines and cell culture

The human astrocyte (HA) cell line and glioma cell lines LN229 and T98 were donated by the Microbiology Laboratory of Henan Provincial People's Hospital. The glioma cell lines U251 and A172 were purchased from Procell Life Science & Technology Co. Ltd (Wuhan, China). Cells were cultured in DMEM high-sugar medium (Procell, PM150210) containing 10% fetal bovine serum (FBS, Gibco, lot: 10099-141c) and 1% penicillin-streptomycin mixture in a 37 ℃ humidified incubator with 5% carbon dioxide.

### Cell transfection

For transient transfection, cells were seeded in 6-well plates, 100 pmol siRNA was transfected into each well using siRNA-Mate (GenePharma, Shanghai, China), and the transfection efficiency was detected by real-time quantitative polymerase chain reaction (RT-qPCR) at 36h after transfection. Lentivirus targeting *GNG5* (shGNG5) was constructed according to the siRNA sequences with the highest knockdown efficiency (Additional file 1: Table S1, Fig. 7b), while empty sequence lentivirus was constructed as a control (shNC). For stable transfection, serum-free medium containing viral fluid was added when the density after glioma cell seed plate reached 50%, according to the instructions, and replaced with complete medium 24 h later. For stable transfection, when the cell density reached 50%, serum-free medium containing lentivirus was added according to the instructions and replaced with complete medium 24 h later. Medium containing 2 μg/mL puromycin was used to select stably transfected cells.

### RNA isolation and RT-qPCR

Total RNA was collected using Trizol Reagent (Thermo Fisher Scientific, Waltham, MA, USA) and RNA was reverse transcribed using the transcriptor first-strand cDNA synthesis kit (Hoffmann-La Roche, Basel, Switzerland), according to the manufacturer's instructions as described previously [23]. RT-qPCR was conducted with SYBR Green on a 7500HT Fast Realtime System (Applied Biosystems, Foster City, CA, USA). The glyceraldehyde-3-phosphate dehydrogenase (*GAPDH*) was used for the normalization of *GNG5*. The relative fold-change in the expression of *GNG5* was determined by using the relative quantification method. The specific primer sequences used in the reverse transcription experiments are shown in Additional file 2: Table S2 and were purchased from Ribo Bio-Technology (Guangzhou, China).

### MTT assay

Cell proliferation was detected by MTT assay. The transfected cells were seeded into 96 well plates (1000 cells/well). 100 μL serum-free medium containing 10 μL MTT (Solarbio, China) was added to each well and incubated at 37 °C for 4 h. Then the supernatant was removed, and 110 μL DMSO was added. After shaking for 10 min in the dark at room temperature, the absorbance of the solution was measured at 490 nm with a spectrophotometer.

### Scratch wound healing assay

Cells stably transfected with shGNG5 and shNC were cultured in 6-well plates, respectively. Three replicate wells per cell were performed for parallel experiments. When cells grew to 90% confluence, each well was scratched with a 200 μL sterile pipette tip and then washed 3 times with 1× PBS to remove detached cells. Each well was added with an appropriate amount of fresh serum-free medium and placed in a 37 °C incubator for 24 h. Photographs of the same area of the wound were taken under 40× magnification conditions using a phase-contrast microscope.

### Transwell migration assay

200 μL serum-free medium containing 2 × 10^4^ transfected cells were seeded into 24 well transwell upper chambers (Corning, USA), respectively. 600 μL of complete medium with 10% FBS was added to the lower chamber. After incubation for 24 h, the non migrated cells on the surface of the upper chamber were erased, then the upper chamber was fixed with 4% paraformaldehyde for 30 min at room temperature and stained with 1% crystal violet for 5 min. The staining results were observed under a microscope and five fields were randomly selected to count the cells. All assays were independently performed in triplicate.

### Colony-forming assay

Transfected cells were resuspended and seeded in 6-well plates (200 cells/well) and cultured in complete medium for 10 days. Cell colonies were fixed using 4% paraformaldehyde for 30 min at room temperature and stained using 1% crystal violet for 5 min. Then, cell colonies were observed under a microscope and imaged, and the number of cell colonies was counted using ImageJ software (Version 1.52).

### Determination of cell cycle distribution

The transfected cells were collected and fixed using ice-cold 70% ethanol for 24 h. According to the cell cycle detection kit instructions (SevenSea Pharmatech, China), cells were then centrifuged and washed with sterile PBS, incubated with 500 μL of staining solution containing 12.5 propidium iodide and 10 μL RNase A for 30 min at 37 °C in the dark. Then, cell cycle distribution was analyzed using a FACSCalibur flow cytometer (BD Biosciences).

### Western blot

Total protein was prepared from transfected cells using RIPA buffer with a proteinase inhibitor (EpiZyme, China). The lysates were incubated on ice for 15 min and then centrifuged at 12,000 rpm for 15 min at 4 °C, and the protein concentration was measured by a BCA kit (Boster, Wuhan, China). Equal quantities of protein were electrophoresed through a 12.5% sodium dodecyl sulfate-polyacrylamide gel and transferred to PVDF membranes (Millipore, Billerica, MA). The membranes were blocked and then probed with the appropriate primary antibody, such as *VCAM1* (1:250, Proteintech, USA), *ICAM1* (1:1000, Cell Signaling Technology, USA), *CDH2* (1:800, Cell Signaling Technology, USA), *SDC2* (1:800, Proteintech, USA), and *GAPDH* (1:5000, Bioworld Technology, USA), overnight at 4 °C. Subsequently, the membranes were incubated with the goat anti-Rabbit IgG secondary antibody (EarthOx Biotechnology) at room temperature for 1 h. The protein blots were developed using a chemiluminescence reagent (ECL) kit (Beyotime Biotechnology).

### Immunochemical staining

For immunohistochemical (IHC) staining, tissue sections of 4 μm thickness were placed in xylene and graded alcohols for deparaffinization and hydration. Heat-induced antigen retrieval was performed in EDTA (PH 8.0) buffer using a microwave oven for 15 min. Blocking was done with 10% goat serum to reduce nonspecific staining. The appropriate amount of primary antibody working solution was then dropped onto the sections and incubated overnight at 4 ℃. Primary antibodies included *GNG5* (1:100, Novus Biologicals, USA) and *IDH1 R132H* (Zhongshan Jinqiao, China). The staining results were observed under a light microscope, and ten 40× visual fields were randomly selected and photographed. The IHC results were analyzed using ImageProPlus software (version 6.0). For cellular immunofluorescence staining, transfected cells were resuspended and seeded in 24 well plates placed with round coverslips (Solarbio, China). Cells were fixed using 4% paraformaldehyde after 24 h and subsequently permeabilized using 0.5% Triton X-100. Primary antibody Ki67 (1:100, abbkine, China) was added onto the slides and incubated overnight at 4 ℃. DyLight 594 IgG (1:100, abbkine, China) was added onto the slides as a fluorescent secondary antibody for 2h at room temperature. DAPI was then used for nucleus staining, and the staining results were observed and imaged under a fluorescence microscope.

### In vivo tumor formation assay

Four week old male nude mice were purchased from the Charles River Animal Experimental Center (ZheJiang, China), and U251 cells stably transfected with shGNG5 and shNC were collected, respectively. The cell concentration was resuspended to 2 × 10^7^ / mL and then 100 μL of cell suspension was subcutaneously injected into the root of the right thigh of each nude mouse. The tumor sizes were measured every five days starting the second week. Tumor volume was calculated by the formula: volume = (longest diameter × shortest diameter^2^) /2 [24]. The mice were sacrificed after 5 weeks, and the tumors were removed and weighed. The protocol was in accordance with the regulations of the animal ethics committee of Harbin Medical University.

### Statistical analysis

We used R (v3.5.1) for statistical analysis. The Wilcoxon rank-sum or Kruskal test was used to analyze the correlation between the expression level of *GNG5* and clinical data of glioma patients. A Cox's regression model and the Kaplan-Meier method were used to analyze the relationship between the expression level of GNG5 and the patients' OS. COX test was used for univariate and multivariate analysis to reveal the risk factors affecting glioma prognosis. A Mann-Whitney test, Chi-square or Fisher's exact test was performed to analyze the different expression of *GNG5* between two groups using the Graphpad Prism 8.0 software.

## Results

### Clinical characteristics of the patients involved in the study

We collected 40 glioma samples, including 19 males and 21 females, and 8 cases with WHO grade II, for a total of 10 and 22 patients with WHO grade III and IV, respectively (Table 1). Furthermore, we used data from the CGGA database that contains a large number of glioma gene expression profiles and a vast amount of clinical information data to further analyze the relationship between *GNG5* expression and the clinical features of glioma. RNA sequencing data of 749 patients, including 442 males and 307 females, with complete clinical information, were screened from 1018 samples based on the CGGA database. In total, we analyzed 502, 222, and 25 cases of primary, recurrent, and secondary gliomas, respectively. An analysis of the pathological results showed that there were 218, 240, and 291 cases with WHO grade II, grade III, and grade IV gliomas, respectively. In addition, 40.28 % of the patients (n = 410) showed mutations in *IDH*, and 33.30 % patients (n = 339) were *IDH* wildtype. Only 15.23 % of patients (n = 155) showed a 1p19q co-deletion, whereas 58.35 % (n = 594) did not have this genotype. These results are summarized in Additional file 3: Table S3.

**Table 1:** Clinical information of 40 glioma samples

	Glioma patients N (%)	Group		*P* value
		High *GNG5*	Low *GNG5*	
Gender				0.2049
Female	21 (52.5%)	13	8	
Male	19 (47.5%)	7	12	
Clinical stage				< 0.001*
WHO II	8 (20%)	0	8	
WHO III	10 (25%)	0	10	
WHO IV	22 (55%)	20	2	
Age (years)	47.15 ± 14.49			0.5273
<= 47.15	21 (52.5%)	9	12	
> 47.15	19 (47.5%)	11	8	
Staining of IDH1 R132H				0.0309*
Positive	18 (45%)	5	13	
Negative	22 (55%)	14	8	

High or low is divided by the median value. Chi-square or Fisher's exact test. *P < 0.05 was considered statistically significant

### GNG5 is highly expressed in gliomas

**Fig. 1:**
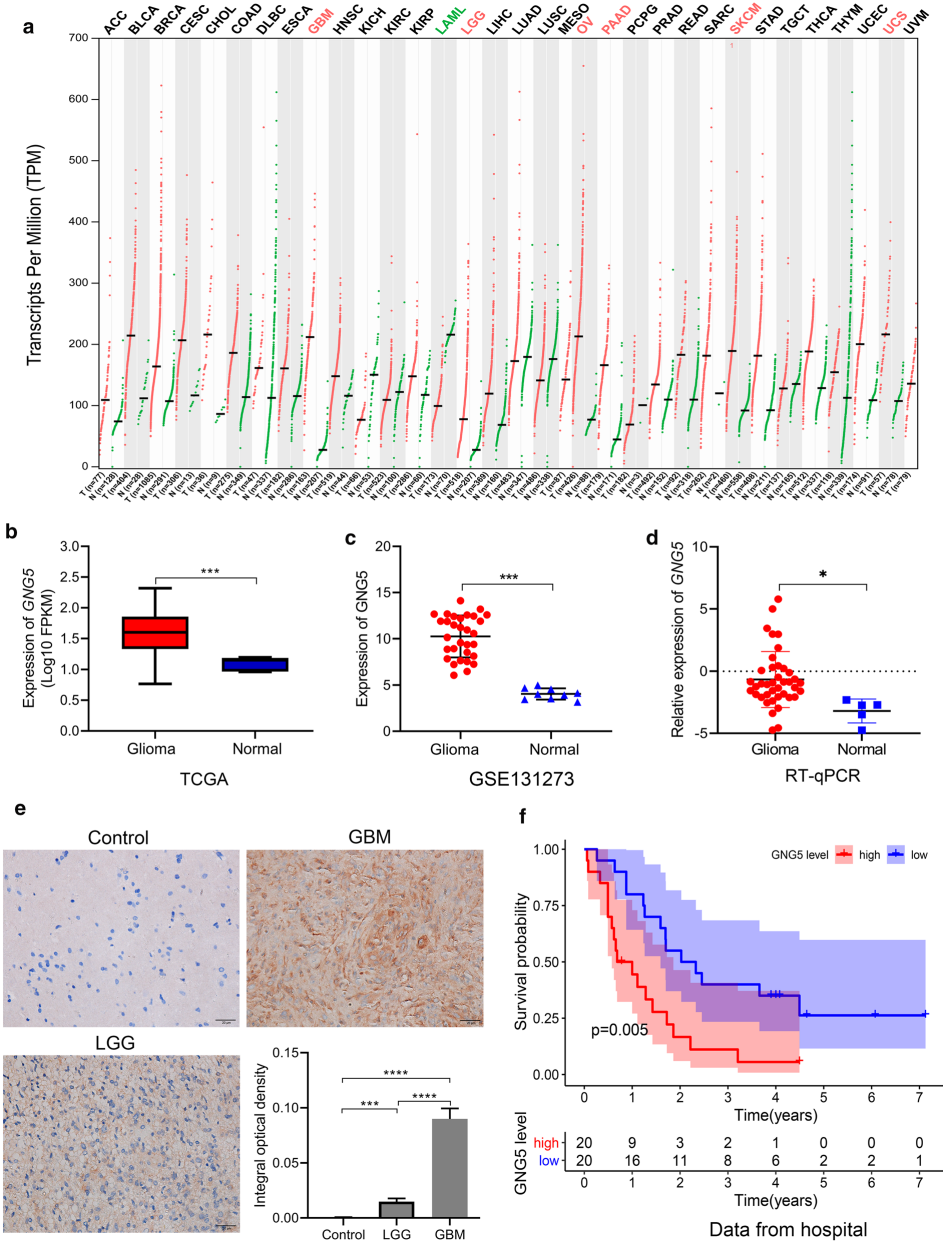
*GNG5* is highly expressed in gliomas and associated with prognosis. **a** Expression of *GNG5* in different types of tumors in GEPIA, red, and green represent significant differences. Red represents a high expression of *GNG5* in tumors, and the green represents a low expression of *GNG5* in tumors; **b**–**d** expression level of *GNG5* in gliomas and normal tissues based on TCGA (**b**), GSE131273 (**c**), and the result of RT-qPCR (**d**); **e**: Results of immunohistochemical staining and statistical analysis of GNG5 in glioma and control samples; **f**: survival analysis based on follow-up results of clinical samples. ****P < 0.0001, ***P < 0.001, *P < 0.05

We analyzed *GNG5* expression in different tumors and control groups using GEPIA, including 9,663 samples from 33 tumors and 5540 corresponding control samples. *GNG5* expression was elevated in tumor tissues including glioblastoma (GBM), lower-grade glioma (LGG), ovarian serous cystadenocarcinoma (OV), pancreatic adenocarcinoma (PAAD), skin cutaneous melanoma (SKCM), and uterine carcinosarcoma (UCS), and showed significantly lower expression in acute myeloid leukemia (LAML) relative to the control group (Fig. 1a). These findings suggest that *GNG5* is highly expressed in a variety of tumors. To further reveal the expression of *GNG5* in gliomas, we analyzed the expression level of *GNG5* in glioma and normal brain tissues based on transcriptome data in TCGA database and chip data in GSE131273 dataset, respectively. And the results showed that *GNG5* expression was significantly higher in gliomas relative to normal brain tissue (Fig. 1b, c, *P* < 0.001), which was consistent with the results of RT-qPCR (Fig. 1D,* P* < 0.05). Furthermore, IHC staining results based on clinical samples showed that the expression level of GNG5 in gliomas was significantly higher than that in control groups and higher in high-grade gliomas than in low-grade gliomas (Fig. 1e), and this result was also confirmed in the HPA database (Additional file 4: Figure S1A). The above results were validated by different databases and clinical samples from transcriptome and protein levels, revealing that *GNG5* is significantly highly expressed in gliomas.

### High expression of GNG5 is a predictor of poor prognosis

**Fig. 2:**
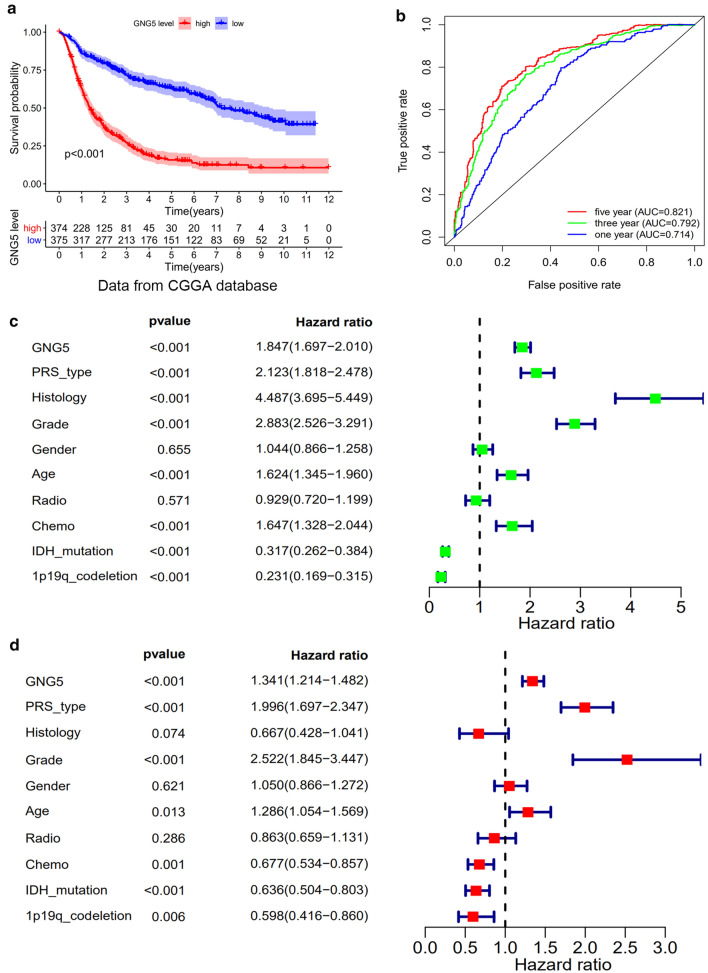
Correlation between *GNG5* and prognosis of glioma patients. **a** The Kaplan–Meier survival curve reveals the high expression of *GNG5* leads to a poor prognosis in gliomas based on CGGA database. **b** The ROC curve shows the good diagnosis value of *GNG5* in gliomas. **c** Univariate regression analysis of prognostic in patients with glioma; **d** multivariate analysis of prognostic in patients with glioma

Analysis of follow-up results of 40 glioma patients showed that patients with high expression of *GNG5* had shorter OS (Fig. 1f, *P* < 0.05), and this result was confirmed in multiple datasets containing large samples. The average follow-up time for the 749 patients whose data for survival analysis is included in this study was 3.22 years. The results showed that high expression of *GNG5* is significantly correlated with reduced survival of glioma patients (Fig. 2a, *P* < 0.001); these results were verified based on TCGA database and GSE53733 dataset (Additional file 4: Figure S1B, C). Moreover, a time-dependent receiver operated characteristic (ROC) analysis showed that the values for area under the ROC curve (AUC) were 0.714, 0.792, and 0.821 for one, three, and five-year OS, respectively (Fig. 2b). Therefore, our results indicate that *GNG5* may serve as a biomarker for glioma patients, especially for the 5-year OS group.

The results of a univariate analysis suggest that the high expression level of *GNG5* in glioma patients is associated with reduced OS, primary recurrence or secondary (PRS) type, histological type, pathological grade, age, chemotherapy status, presence of mutations in the *IDH* gene, and a 1p/19q co-deletion (Fig. 2c). Additionally, the multivariate analysis results indicate that the expression of *GNG5*, PRS type, pathological grade, age, chemotherapy status, presence of mutations in the *IDH* gene, and 1p/9q co-deletion were independently correlated with OS (Fig. 2d). The above data indicate *GNG5* may serve as a prognostic factor and increased *GNG5* expression is associated with poor OS.

### Relationship between GNG5 and the underlying molecular and clinical characteristics in glioma patients

**Fig. 3:**
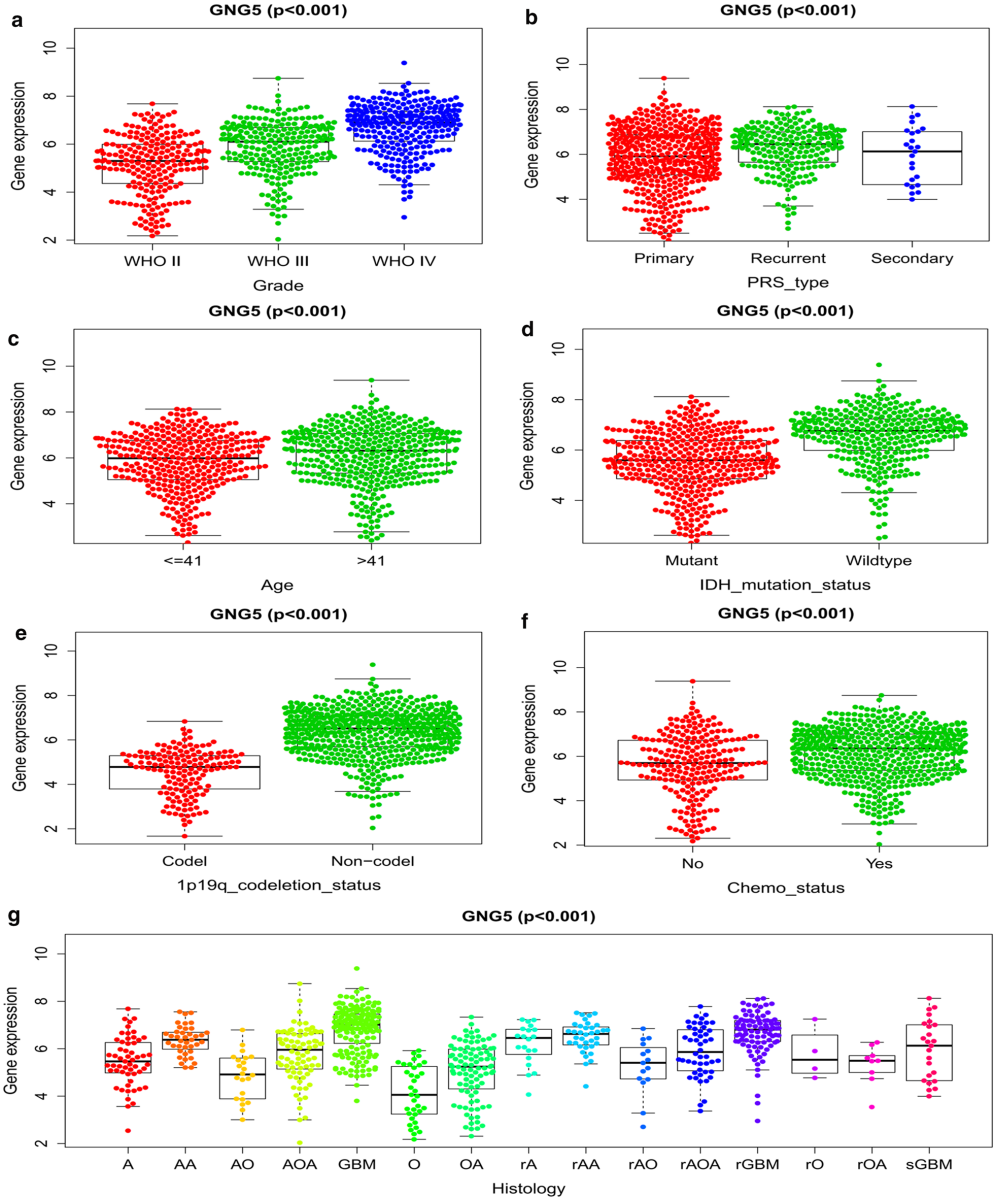
Expression characteristics of *GNG5* in gliomas based on CGGA

To clarify the correlation between highly expressed *GNG5* in gliomas and clinicopathological features, we first analyzed the clinicopathological information of 40 glioma patients and found that the expression level of *GNG5* was significantly correlated with the pathological grade of gliomas (Table 1). To explore whether the expression level of *GNG5* is also associated with more clinicopathological features in a large dataset, we further analyzed the relationship between *GNG5* expression level in the 749 samples from the CGGA database and the various tumor subtypes, their pathological classification, molecular classification, tumor treatment, and the age of the patients. The results indicated that the *GNG5* expression level in gliomas was significantly correlated with the pathological grade and the age of the patients (Fig. 3a, c, *P* < 0.001). The average level of *GNG5* expression in tissue from patients with recurrent gliomas was higher than in patients with primary and secondary tumors (Fig. 3b, *P* < 0.001). Similar results were found based on correlation analysis between *GNG5* expression level in gliomas and different pathological subtypes, such as the expression levels of *GNG5* in recurrent astrocytoma, recurrent anaplastic astrocytoma, and recurrent anaplastic oligodendroglioma were higher than that in corresponding primary pathological subtypes, as shown in Fig. 3g.

Reports from research conducted previously show that patients with *IDH* mutations or a 1p/19q co-deletion had longer survival and better prognosis [25]. Interestingly, we found that the expression level of *GNG5* in patients with mutations in the *IDH* gene was lower than in patients with wildtype *IDH* (Fig. 3d). Similarly, patients with a 1p/19q co-deletion had significantly lower expression of *GNG5* than in patients without the co-deletion (Fig. 3e). Furthermore, to visualize the association between *GNG5* expression and *IDH* mutation in clinical samples, we performed IHC staining on clinical samples, which showed a significant correlation between IDH mutation and GNG5 expression levels in gliomas (Table 1, Additional file 5: Figure S2). Together, these results further indirectly reveal that the expression of *GNG5* correlates with the prognosis of glioma patients. When a grouping of patients based on whether they received chemotherapy or not was performed, we found that patients who received chemotherapy showed an upregulation of *GNG5* expression relative to those who did not receive chemotherapy (Fig. 3f). However, radiotherapy did not affect *GNG5* expression (data was not shown). One explanation for this observation may be that the surgical resection of the tumor and subsequent sequencing data analysis are from samples collected from patients before they underwent chemotherapy. Taken together, these results indicate that a correlation exists between *GNG5* expression and the molecular and clinical characteristics of glioma.

### Co-expression analysis of GNG5

**Fig. 4:**
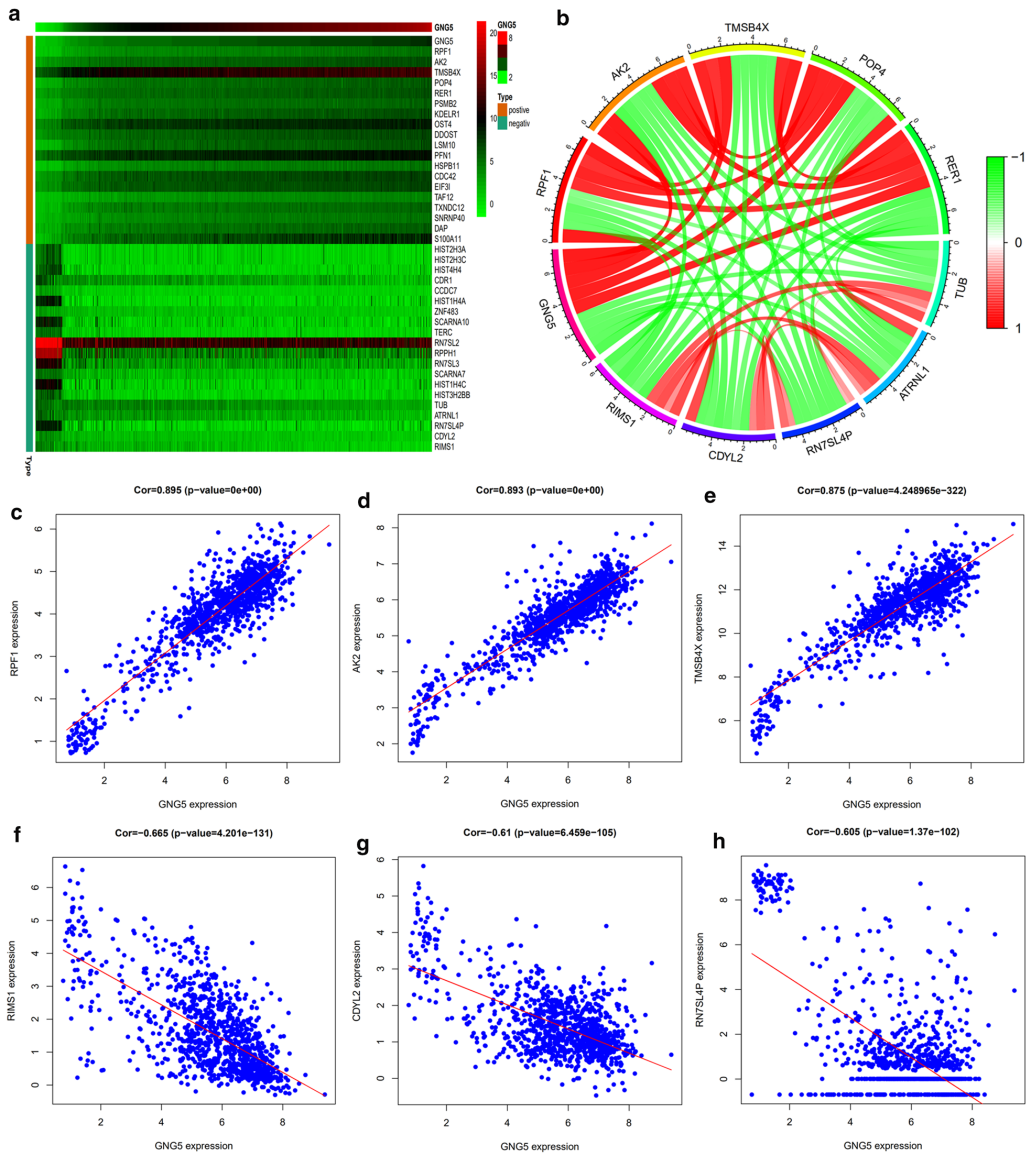
Co-expression analysis of *GNG5*. **a** Heatmap of the top 20 genes associated with *GNG5*; **b** Circos diagram shows the five most significant genes of positive and negative correlating with *GNG5*; **c–h** The correlation between *GNG5* and *RPF1* (**c**), *AK2* (**d**), *TMSB4X* (**e**), *RIMS1* (**f**), *CDYL2* (**g**), *RN7SL4P* (**h**)

To further explore the function of *GNG5*, the limma package (Bioconductor) was used in the R statistical software to analyze the coexpression genes with *GNG5*. We found 4517 genes associated with *GNG5* (correlation coefficient (Cor) > 0.5, *P* < 0.001), of which 29 genes were negatively correlated with *GNG5* and a total of 4488 showed a positive correlation. A heatmap of the top 20 genes associated with *GNG5* is shown in Fig. 4a and the five genes showing significant positive or negative correlation with *GNG5* is shown in Fig. 4b. The five genes that showed positive correlation with *GNG5* expression were *RPF1* (Cor = 0.895, *P* = 0.000), *AK2* (Cor = 0.893, *P* = 0.000), *TMSB4X* (Cor = 0.875, *P* = 4.249 x 10^−322^), *POP4* (Cor = 0.874, *P* = 4.247 × 10^-320^) and *RER1* (Cor = 0.866, *P* = 1.400 × 10^-307^) (Fig. 4c–e and Additional file 6: Figure S3A, B), while the top five genes that were negatively correlated with *GNG5* expression were *RIMS1* (Cor = − 0.665, *P* = 4.201 × 10^−131^), *CDYL2* (Cor = − 0.61, *P* = 6.459 × 10^−105^), *RN7SL4P* (Cor = − 0.605, *P* = 1.370 × 10^−102^), *ATRNL1* (Cor = − 0.595, *P* = 1.953 × 10^−98^) and *TUB* (Cor = − 0.589, *P* = 3.049 × 10^−96^) (Fig. 4f–h and Additional file 6: Figure S3C, D). Thus, *GNG5* expression is correlated with various genes in gliomas.

### GNG5 regulates signaling pathways, including cell adhesion molecules in glioma

**Fig. 5:**
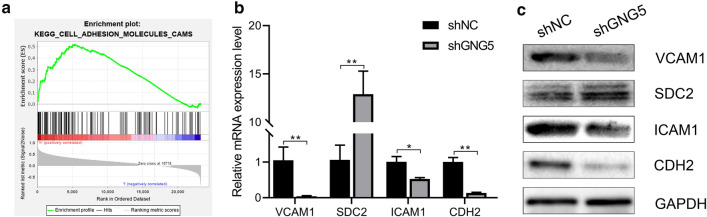
GNG5 is involved in regulating cell adhesion molecules pathways. **a** GSEA enrichment analysis results of the cell adhesion molecules; **b** relative mRNA expression levels of *VCAM1*, *SDC2, ICAM1* and *CDH2*; **c** western blot result of VCAM1, SDC2, ICAM1 and CDH2 in U251 cell line with shGNG5 and shNC. **P < 0.01, *P < 0.05

GSEA was used to identify *GNG5* related signaling pathways involved in gliomas. A *P*<0.05 and a false discovery rate (FDR) < 0.25 represented a significant enrichment in the results (in the enrichment of MSigDB Collection). Five pathways, including those involving the ECM-receptor interaction, focal adhesion, cell adhesion molecules, toll-like receptor signaling pathway, and nod-like receptor signaling pathway, showed significantly differential enrichment in samples from patients showing the *GNG5* high expression phenotype based on NES, NOM *P*-values, and FDR values (Fig. 5a, Additional file 6: S3E–H; Table 2), indicating a potential role for *GNG5* in the development of glioma. Since cell adhesion molecules have an essential role in the proliferation and migration of tumors, to clarify whether glioma cells are involved in cell adhesion molecule regulation, we performed in vitro experiments. We found that decreasing the expression level of *GNG5* in glioma cells could significantly affect the expression of *VCAM1*, *ICAM1*, *CDH2*, and *SDC2*, which are key molecules of cell adhesion molecules (Fig. 5b, c). Therefore we are more confident that *GNG5* can exert its biological effects by regulating different signaling pathways including cell adhesion molecules in glioma.

**Table 2:** Signaling pathways enriched in high expression of *GNG5*.

Gene set name	NES	NOM *P*-value	FDR q-value
KEGG_ECM_RECEPTOR_INTERACTION	1.85	0.008	0.1
KEGG_FOCAL_ADHESION	1.81	0.004	0.076
KEGG_CELL_ADHESION_MOLECULES_CAMS	1.77	0.026	0.096
KEGG_TOLL_LIKE_RECEPTOR_SIGNALING_PATHWAY	1.76	0.002	0.092
KEGG_NOD_LIKE_RECEPTOR_SIGNALING_PATHWAY	1.75	0.016	0.093

### GNG5 is related to the immune microenvironment

**Fig. 6:**
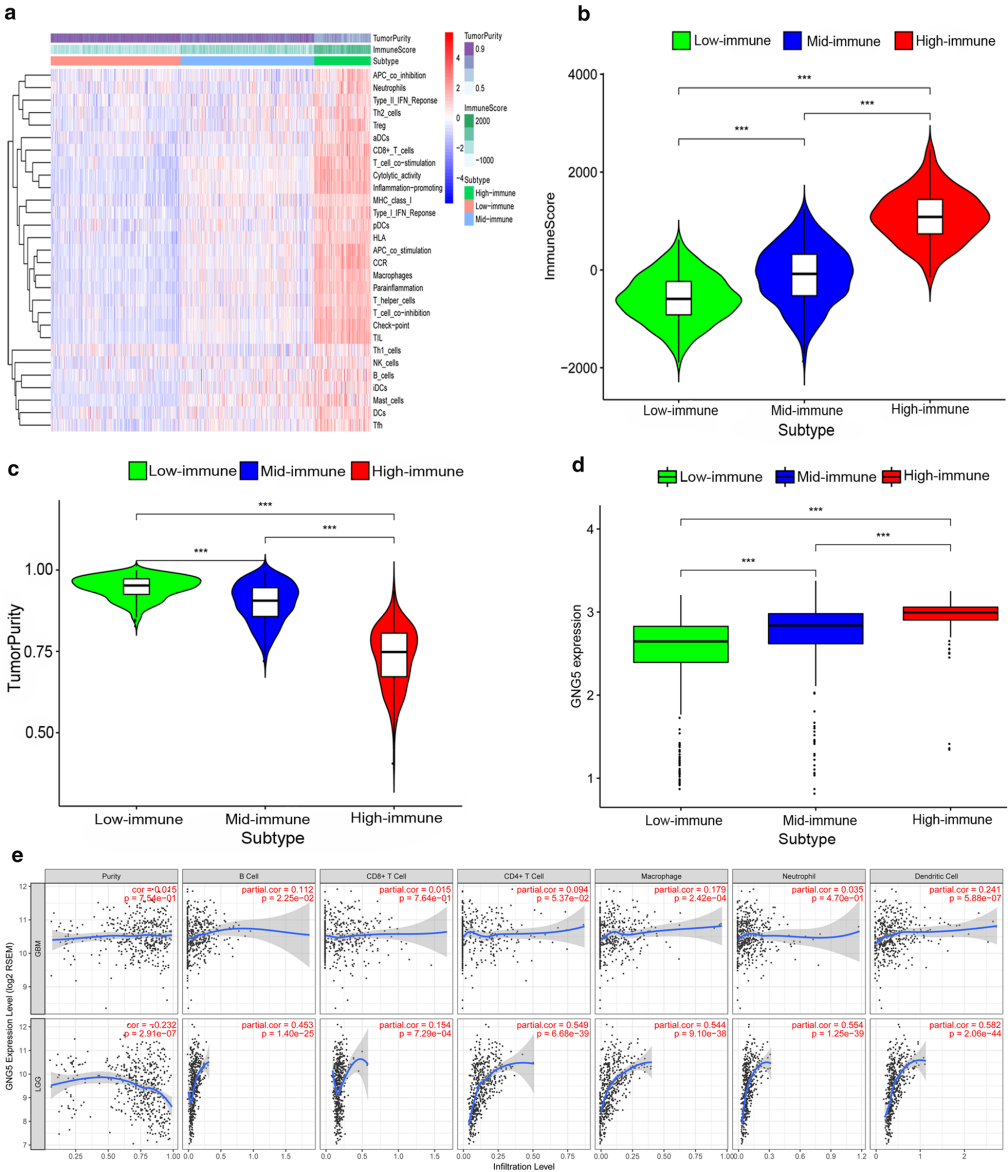
Correlation analysis of *GNG5* and immune microenvironment. **a** Cluster analysis and immune activity quantification based on ssGSEA and ESTIMATE; **b** The difference of immune score between groups in glioma; **c** Relationship between tumor purity and immunity in glioma; **d** Relationship between *GNG5* immunity; **e** Correlation between *GNG5* and immune cells in gliomas based on TIMER. ***P < 0.001

Next, we explored whether there is a correlation between *GNG5* expression and the tumor immune microenvironment. We analyzed 29 immune-system related gene sets characterizing different types and functions of immune cells (Additional file 7: Table S4). Based on data from the CGGA database, ssGSEA was used to quantify and hierarchically cluster immune cells in tumor samples into three groups. According to the clustering heat map of immune cell gene sets in the three groups, a high immunity group (high-immune), medium immunity group (mid-immune), and low immune activity group (low-immune) were defined (Fig. 6a). Moreover, the high-immune group had a significantly higher immune score than that of the low-immune group, though the tumor purity showed the opposite characteristics (Fig. 6b, c). Interestingly, we found that the expression of *GNG5* was significantly increased in the high-immune group, and decreased in the low-immune group (*P *< 0.001, Fig. 6d). Further, we used TIMER to analyze the relationship between *GNG5* expression and infiltration abundance of six immune cells (B cells, CD4+ T cells, CD8+ T cells, neutrophils, macrophages, and dendritic cells) after purity correction in glioma [22, 26]. We found that *GNG5* expression was positively correlated with the infiltrating abundance of CD8+T cell (Cor = 0.154 , *P* = 7.29 × 10^−4^), B Cell (Cor = 0.453 , *P* = 1.40 × 10^−25^), macrophages (Cor = 0.544, *P* = 9.10 × 10^−38^), CD4+T cells (Cor = 0.549, *P* = 6.68 × 10^−39^), neutrophils (Cor = 0.554, *P* = 1.25 × 10^−39^), dendritic cell (Cor = 0.582, *P* = 2.06 × 10^−44^) in LGG. B Cell (Cor = 0.112, *P* = 0.022), macrophages (Cor = 0.179, *P* = 0.000), and dendritic cells (Cor = 0.241, *P* = 5.88 × 10^−7^) were corrected with *GNG5* expression in GBM (Fig. 6e). These results suggest that *GNG5* may be a potential factor influencing the immune microenvironment in glioma.

### Downregulated GNG5 could inhibit glioma cell proliferation and migration

**Fig. 7:**
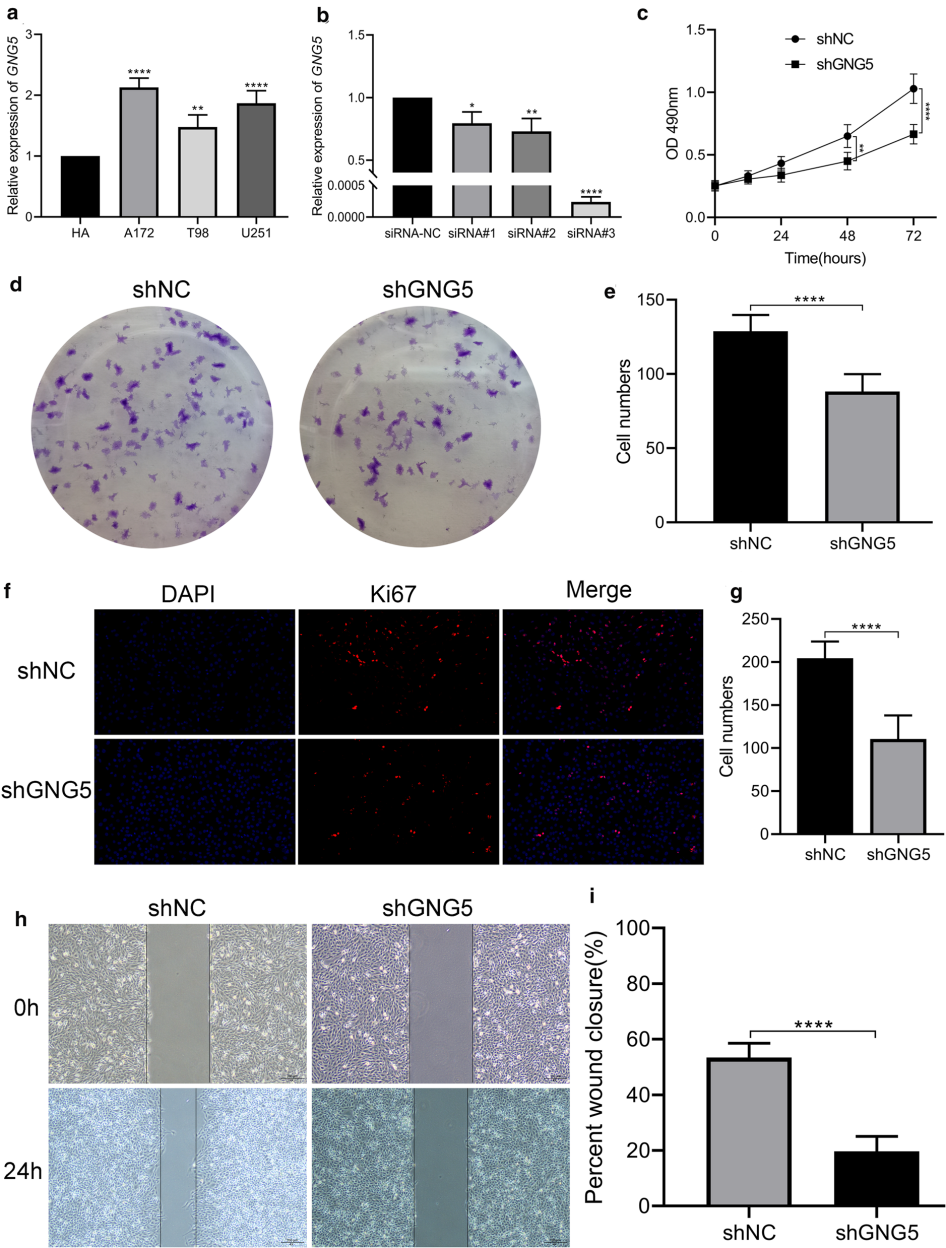
Knockdown of GNG5 expression in glioma cells can affect the functional phenotype of glioma cells. **a** The expression levels of GNG5 in different glioma cell lines and human astrocytes (HA) were analyzed based on RT-qPCR; **b** Efficiency comparison of different siRNA sequences for targeted knockdown of GNG5 in U251 cell line; **c** MTT assay results of U251 cell lines transfected with shGNG5 and shNC; **d** Clonogenic results of U251 cell lines transfected with shGNG5 and shNC; **e** analysis of clonogenic results; **f** Ki67 immunofluorescence staining results of U251 cell lines transfected with shGNG5 and shNC; **g** Analysis of Ki67 staining results; **h** Results of scratch healing ability assay of U251 cell lines transfected with shGNG5 and shNC; **i** Analysis of scratch healing capacity test results. ****P < 0.0001, ***P < 0.001, **P < 0.01, *P < 0.05

**Fig. 8:**
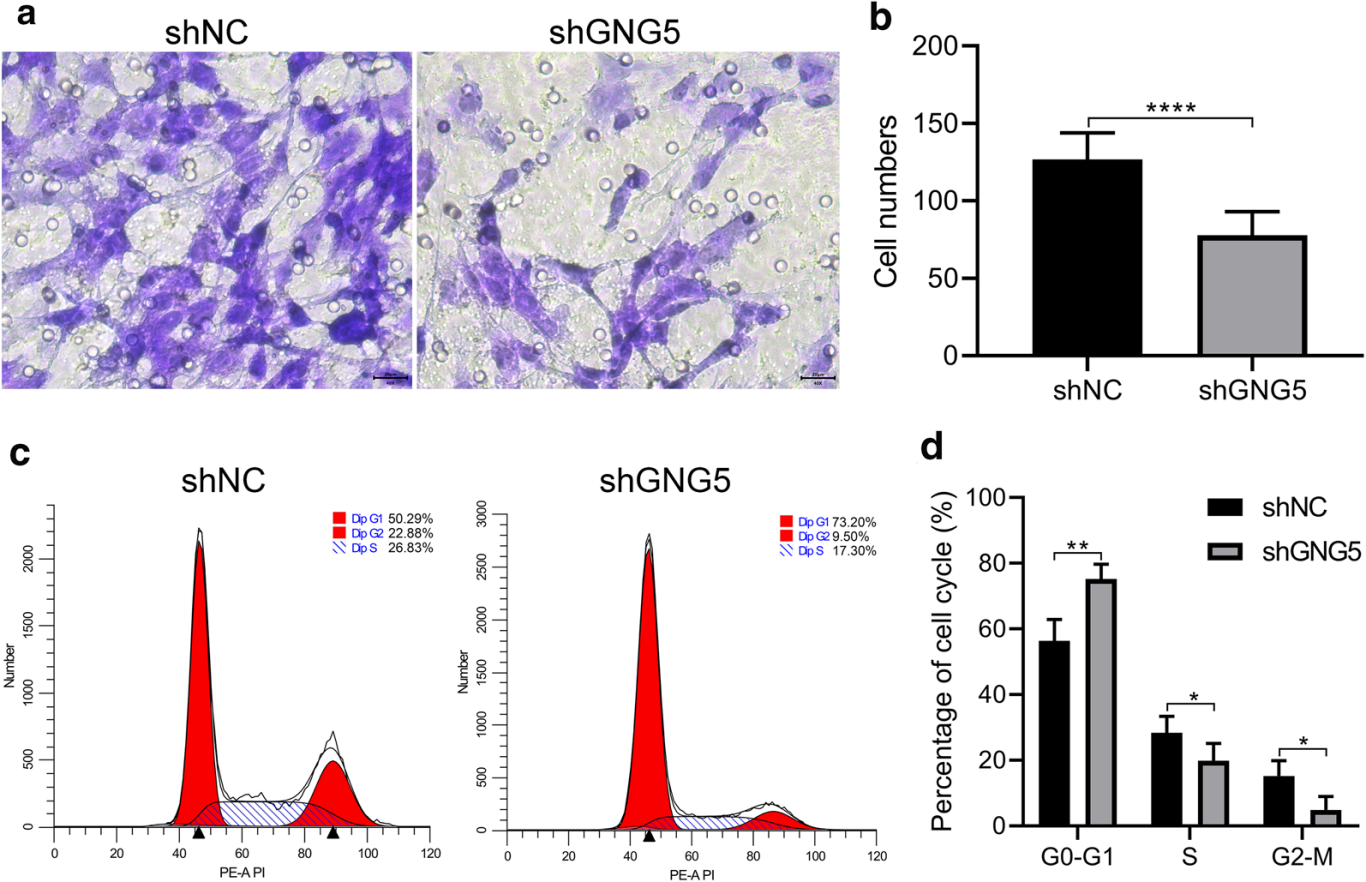
Transwell and cell cycle distribution assay results. **a** Results of Transwell experiments with U251 cell lines transfected with shGNG5 and shNC; **b** Analysis of Transwell assay results; **c** Cell cycle distribution assay results of U251 cell lines transfected with shGNG5 and shNC; **d** Analysis of cell cycle distribution assay results. **P < 0.01, *P < 0.05

**Fig. 9:**
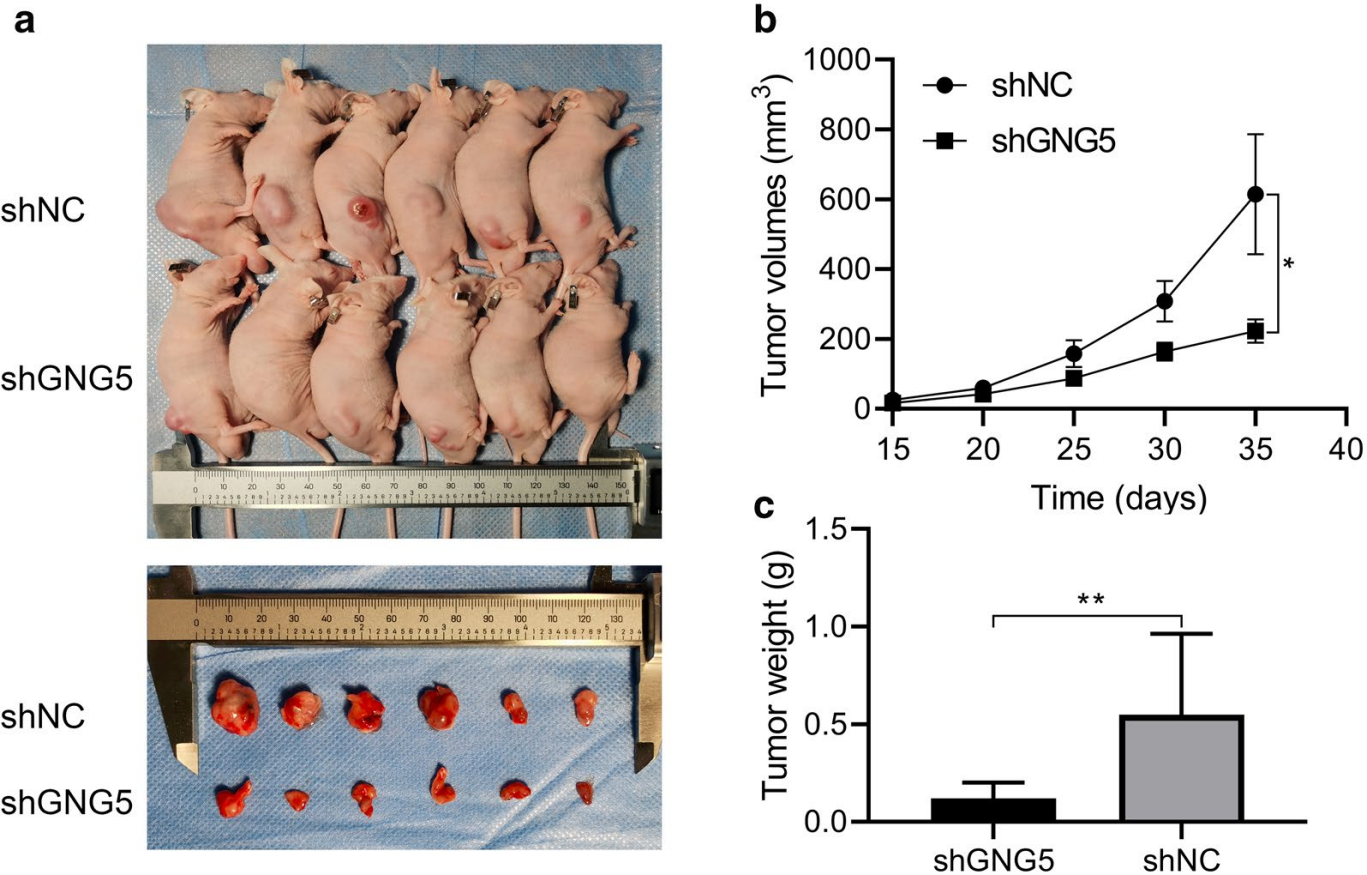
Knockdown of GNG5 can inhibit glioma growth based on in vivo experiments. **a** Tumor histomorphology; **b** Comparison of changes in tumor tissue volume between experimental and control groups; **c** Bar graphs comparing tumor tissue weights between experimental and control groups. **P < 0.01, *P < 0.05

RT-qPCR results showed that the expression level of *GNG5* in many kinds of glioma cells was significantly higher than that in HA cells (Fig. 7a). To explore the role of *GNG5* in gliomagenesis, we constructed and screened siRNA sequences targeting *GNG5* (Fig. 7b, Additional file 1: Table S1). To observe the effect of downregulated *GNG5* on migration and proliferation of tumor cells, we established a U251 glioma cell line with stable knockdown of *GNG5*. MTT assay indicated that the cell proliferation ability decreased obviously after reducing the expression of *GNG5* (Fig. 7c), which was similarly confirmed by cell clonogenic assay (Fig. 7d, e) and Ki67 IF staining (Fig. 7f, g). In addition, we also found that the migration ability of tumor cells was reduced after the reduction of *GNG5* expression and was significantly different from that of the control group (Fig. 7h, i *P *< 0.001). Compared with the control group, the number of glioma cells migrated significantly decreased in shGNG5 group (Fig. 8a, b). Further, we found that knockdown of *GNG5* could arrest the cells in the G1 phase (Fig. 8c, d), suggesting that *GNG5* may play a biological function by participating in the cell cycle process. Moreover, subcutaneous tumorigenesis experiments showed that downregulation of *GNG5* could significantly inhibit the growth of glioma tissues (Fig. 9, *P* < 0.05). The results of in vivo and in vitro experiments indicated that *GNG5* could participate in the malignant progression of gliomas by participating in the proliferation and migration of gliomas.

## Discussion

G-proteins consisting of α, β and γ subunits are also known as GTP binding proteins, which mediate the transduction of a variety of hormones and neurotransmitters across the membrane and subsequently trigger a series of physiological and biochemical reactions in cells [27, 28]. G-proteins regulate basic life processes such as cell metabolism, secretion, growth, proliferation, differentiation, distortion, pathological changes, and cell death [4]. *GNG5*, a subunit of G-protein, has been reported to promote the proliferation and migration of tumor cells [29]. However, to date, few studies have reported the role and mechanism of action for *GNG5* in tumors.

To explore the expression patterns of *GNG5* in various tumors, we searched for *GNG5* in the GEPIA database. We found that *GNG5* was significantly highly expressed in a variety of cancers, including gliomas (Fig. 1a). To further understand the role of *GNG5* in tumors, we focused on its role in glioma. We obtained *GNG5* expression data from TCGA and GEO databases and clinical samples. Our results showed that *GNG5* expression was increased in glioma based on data from sequencing, microarrays, and qPCR data (Fig. 1b–d). Previous studies have suggested that *GNG5* shows elevated expression in endometrial cancer and in infiltrating ductal carcinoma of the breast [15, 16]. Furthermore, *POP4*, a *GNG5* coexpressing promoter gene, is highly expressed in prostate cancer cells, while *RIMS1*, a *GNG5* coexpressing suppressor gene, is lowly expressed in craniopharyngioma [30, 31]. Moreover, we validated the expression pattern of *GNG5* protein in glioma samples obtained from clinic and HPA databases, and a high level of *GNG5* was observed in glioma (Fig. 1e, Additional file 4: Figure S1A). Moreover, our follow-up results suggested that *GNG5* highly expressed in gliomas could significantly decrease patients' OS. Therefore, we hypothesized that *GNG5* might be an oncogene in glioma.

To elucidate in large sample data that *GNG5* is an oncogene and can lead to a poorer prognosis for patients, we obtained clinical information on more than a thousand glioma samples from TCGA, CGGA, and GEO databases. The results show that increased expression of *GNG5* could indeed cause a poor prognosis of glioma patients. Moreover, as shown in Fig. 2b, *GNG5* has a high diagnostic value and can be used as a biomarker to predict glioma prognosis. Additionally, genes coexpressed with *GNG5,* such as *AK2,* are highly expressed in lung cancer and can lead to poor prognosis in patients [32]; besides, *RER1* as a coexpressed gene with *GNG5*, could promote the progression of pancreatic cancer and reduce the survival rate of patients [33]. Although the high expression of GNG5 in glioma could reduce patients' OS rate, whether the expression of *GNG5* is necessarily correlated with such a prognosis for glioma patients was further examined. Results from univariate and multivariate analyses showed that increased expression of *GNG5* is an independent risk factor for glioma patients. The above results indicate that *GNG5* is indeed an oncogene in gliomas and is predictive of a worse prognosis for patients. Furthermore, we found that *GNG5* expression levels are closely related to the clinical features associated with glioma prognosis, especially with the pathological grade and primary recurrence status of glioma (Fig. 3, Table 1). However, the results based on clinical data were not as significant in the correlation analysis between the expression level of *GNG5* and age in the CGGA database, possibly due to the small sample size. Some studies have reported that glioma patients with a mutation in the *IDH* gene and a 1p/19q co-deletion have a better prognosis [34, 35]. As we predicted, we found that the average expression level of *GNG5* was lower in samples from patients with mutations in the *IDH* gene and those with a 1p/19q co-deletion. Moreover, since approximately 90% of *IDH* mutations occur in the *IDH1* gene and more than 85% of mutations occur at R132H [25, 36], through IHC analysis, we found that GNG5 was significantly associated with IDH mutation at the protein level. The above results reveal *GNG5* as an oncogene in gliomas from different perspectives and portend a worse prognosis for patients. However, the mechanism explaining how *GNG5* leads to a poor prognosis for glioma patients is yet to be elucidated.

We next undertook a GSEA to understand the functioning of *GNG5* in glioma. We found that high expression of *GNG5* promotes the activation of a series of cancer-related signaling pathways (Fig. 6). Similar interactions have been described previously; for example, the ECM-receptor interaction is involved in the proliferation and invasion of cancer cells [37, 38], and focal adhesion is involved in the metastasis and invasion of cancer cells [39]. Additionally, the toll-like receptor promotes the immune evasion of glioma by down-regulating the MHC II molecules in microglia [40], and cell adhesion molecules and the nod-like receptor play a significant role in the proliferation, invasion, and metastasis of glioma cells [41–44]. A particular gene may play a role in a variety of signaling pathways in the pathological process of diseases. Used as a tool to reveal the molecular mechanisms underlying diseases, GSEA is widely used and has high reliability. Compared with traditional methods of analysis, GSEA has the advantage of using large sample sizes and can avoid the biases inherent in experimental results caused by artificial threshold setting [17, 45]. Furthermore, based on the results of GSEA, we found that decreasing the expression levels of *GNG5* could significantly affect the expression of key molecules of cell adhesion molecules signaling pathway, such as *VCAM1*, *ICAM1*, *CDH2*, and *SDC2* [47], from the mRNA and protein levels. It is well known that cell adhesion molecules play an essential role in tumors' malignant progression [46]. Moreover, previous studies have shown that downregulation of *VCAM1*, *ICAM1,* and *CDH2* can inhibit cancer cell proliferation and migration [46, 48–50]. In our study, we found that the expression of *VCAM1*, *ICAM1*, *CDH2* was down-regulated, while the expression of *SDC2* was up-regulated after knocking down the expression of *GNG5* in glioma cells. The expression level of *SDC2* was found to be up-regulated after knocking down the expression of *GNG5* in the present study, possibly related to reducing its methylation level. Previous studies have shown that *SDC2* has a higher methylation level in cancer tissues [51, 52], which indirectly supports our hypothesis. These results further illustrate that downregulation of *GNG5* can inhibit glioma cell proliferation and migration through the cell adhesion molecules pathway. Together, we believe that the signaling pathways enriched with *GNG5* in glioma, as revealed in our study, could provide the basis for further investigations into the specific mechanisms underlying *GNG5* functioning in glioma.

Recently, immunotherapy has been shown to be a new treatment strategy for glioma, and genes related to glioma immunity have been reported frequently [53, 54]. However, the association of *GNG5* with glioma immunity has not been reported. Our results suggest that *GNG5* may participate in the malignant progression of glioma through ECM-receptor interaction, toll-like receptor pathway, and the nod-like receptor signaling pathways, which are reported to be related to the immune response process [55, 56]. Therefore, we hypothesized that *GNG5* might be involved in the immune response in glioma. To verify this, we analyzed 29 immune-associated gene sets in glioma samples using ssGSEA and ESTIMATE. The results showed that the expression of *GNG5* in glioma was correlated with immune activity (Fig. 7d). Previous studies have reported a correlation between programmed death-1/ligand-1 (PD-1/PD-L1) and immune activity in tumors in triple-negative breast cancer [19]; Moreover, a large number of studies have shown that ssGSEA and ESTIMATE are highly reliable in the evaluation of tumor immune stratification and immune invasion analysis [21, 57–60]. Furthermore, we analyzed the correlation between the expression of *GNG5* and infiltration level of tumor-infiltrating immune cells using TIMER. The results suggest that *GNG5* is correlated with the infiltration abundance of various immune cells in glioma, especially macrophage, dendritic cells, and neutrophils in low-grade glioma (Fig. 7e). Though there are currently few reports describing the relationship between the G-protein family and cancer immunity, correlations between single genes and cancer immunity have been observed. For example, CD70, which is highly expressed in glioma, can promote macrophage infiltration into a tumor [53], and JAK1 was shown to be positively correlated with the infiltration of various immune cells in breast cancer, such as CD8+ T cells and dendritic cells, etc. [61]. Additionally, LAYN expression was positively correlated with the infiltration of CD4+T and CD8+T cells, macrophages, neutrophils, and dendritic cells in colon and gastric adenocarcinomas [62]. These results indicate that *GNG5* is a highly expressed gene in glioma, is associated with immune activity, and future research could help establish the immune response related to *GNG5* function in glioma.

In order to study the function of *GNG5* in glioma cells in vitro and in vivo, we knocked down the expression level of *GNG5* in glioma cells by cell transfection technology. We found that knockdown of the expression level of GNG5 significantly reduced the metastatic and proliferative abilities of glioma cells. This effect may be related to the involvement of *GNG5* in the cell cycle, as downregulated *GNG5* can significantly arrest glioma cells in the G1 phase. In vivo experiments further confirmed that the growth tendency of tumor tissue could be obviously inhibited by downregulated *GNG5*.

Although we used bioinformatics analysis and large datasets to uncover the role of *GNG5* in glioma, our study has a few limitations. First, as extensive clinical sample information in the public databases is not always available, it is difficult to comprehensively evaluate the correlation between *GNG5* and the clinical features of glioma patients. However, it is difficult to avoid the problem of the lack of clinical sample information in public databases, as this data is collated from different research centers. Second, through comprehensive biological analysis, we found that GNG5 may be involved in a variety of biological processes to affect the prognosis of gliomas, although the in vitro and in vivo experiments involved in this study are only preliminary verification. However, our results confirm the reliability of biological analysis results, which lays a foundation for further study of various mechanisms and elucidating the mechanism of disease in the future.

## Conclusion

Our study shows that *GNG5* is highly expressed in gliomas, and this expression is correlated with various molecular and clinical features of glioma patients. High expression levels of *GNG5* predict a poor prognosis in glioma patients. Decreasing the expression of *GNG5* can inhibit glioma cell proliferation and migration. Additionally, *GNG5* may participate in glioma's pathological progress through the signaling pathways related to cancer, such as cell adhesion molecules signaling pathway. These results indicate that *GNG5* is a novel oncogene in glioma and could provide a potential biomarker for diagnosing and treating gliomas.

## Supplementary Information

**Additional file 1: Table S1.** siRNA sequences targeting *GNG5*.

**Additional file 2: Table S2.** RNA-specific primers sequences.

**Additional file 3: Table S3.** Clinical characteristics of glioma patients based on CGGA RNA-seq data.

**Additional file 4: Figure S1:** Correlation between *GNG5* expression level and overall survival of glioma patients. (A): Immunohistochemical results of GNG5 in glioma and normal tissues based on human protein atlas. LGG: low grade glioma, HGG: high grade glioma; (B): Kaplan-Meier curve based on TCGA database; (C): Differences in *GNG5* expression in patients with different survival periods based on GEO (GSE 53733).

**Additional file 5: Figure S2:** Correlation between IDH1 R132H and GNG5 in glioma samples. (A): Representative results of IDH1 R132H immunohistochemical staining in GNG5 positive tissue; (B): correlation analysis of GNG5 and IDH1 R132H based on the clinical sample. *P = 0.0309.

**Additional file 6: Figure S3:** Co-expression analysis of *GNG5* and GSEA enrichment analysis results. The correlation between *GNG5* and *POP4* (A), *RER1* (B), *ATRNL1* (C), *TUB* (D); GSEA enrichment analysis of the ECM-receptor interaction (E), the focal adhesion (F), the toll-like receptor signaling pathway (G) and the nod-like receptor signaling pathway (H).

**Additional file 7: Table S4.** Gene sets associated with different immune cells.

## Data Availability

The datasets generated and/or analysed during the current study are available in the CGGA database (http://www.cgga.org.cn/), TCGA database (https://portal.gdc.cancer.gov/), and datasets from GEO (https://www.ncbi.nlm.nih.gov/geo/). The data of RT-qPCR and cell function experiments during the current study are available from the corresponding author on reasonable request.

## Abbreviations

AUC: Area under the receiver operated characteristic curve
CGGA: Chinese Glioma Genome Atlas
CNS: Central nervous system
Cor: Correlation coefficient
EMT: Epithelial-mesenchymal transition
FDR: False discovery rate
GAPDH: Glyceraldehyde-3-phosphate dehydrogenase
GBM: Glioblastoma
GEO: Gene Expression Omnibus
GEPIA: Gene Expression Profiling Interactive Analysis
HPA: Human Protein Atlas
IDH: Isocitrate dehydrogenase
IHC: Immunohistochemical
LAML: Acute myeloid leukemia
LGG: Lower-grade glioma
NES: Normalized enrichment scores
NOM: Nominal
OS: Overall survival
OV: Ovarian serous cystadenocarcinoma
PAAD: Pancreatic adenocarcinoma
PD-1/PD-L1: Programmed death-1/ligand-1
PRS: Primary recurrence or secondary
ROC: Receiver operated characteristic
SKCM: Skin cutaneous melanoma
ssGSEA: Single-sample gene set enrichment analysis
TCGA: The Cancer Genome Atlas
TIMER: Tumor IMmune Estimation Resource
UCS: And uterine carcinosarcoma
WHO: World Health Organization
