# Supplementary material for: GNG5 is a novel oncogene associated with cell migration, proliferation, and poor prognosis in glioma
Source: Cancer Cell Int. 2021 Jun 7;21:297. doi: 10.1186/s12935-021-01935-7 (PMC8186147; doi:10.1186/s12935-021-01935-7)
Supplement: Supplementary file 1 — Additional file 1: Table S1. siRNA sequences targeting GNG5. [file 12935_2021_1935_MOESM1_ESM.docx]

Table S1 siRNA sequences targeting *GNG5*.

| siGNG5 | Sequence (5’-3’) |
| --- | --- |
| siRNA#1- forward | CGUCGCCGCUAUGAAGAAATT |
| siRNA#1- reverse | UUUCUUCAUAGCGGCGACGTT |
| siRNA#2- forward | GCAGACUUGAAACAGUUCUTT |
| siRNA#2- reverse | AGAACUGUUUCAAGUCUGCTT |
| siRNA#3 (shGNG5)- forward | GGAGUAUCUUCAAGUACAATT |
| siRNA#3 (shGNG5)- reverse | UUGUACUUGAAGAUACUCCTT |
| siRNA#NC-forward | UUCUCCGAACGUGUCACGUTT |
| siRNA#NC- reverse | ACGUGACACGUUCGGAGAATT |

NC: Negative control
