# Supplementary material for: GNG5 is a novel oncogene associated with cell migration, proliferation, and poor prognosis in glioma
Source: Cancer Cell Int. 2021 Jun 7;21:297. doi: 10.1186/s12935-021-01935-7 (PMC8186147; doi:10.1186/s12935-021-01935-7)
Supplement: Supplementary file 2 — Additional file 2: Table S2. RNA-specific primers sequences. [file 12935_2021_1935_MOESM2_ESM.docx]

Table S2 RNA-specific primers sequences

| Gene | Primers sequences (5’-3’) |
| --- | --- |
| *GNG5*-forward | CGGACTCAACCGCGTAAA |
| *GNG5*-reverse | GGGTCTGAAGGGATTTGTACTT |
| *ICAM1*-forward | AGCTTCGTGTCCTGTATGGC |
| *ICAM1*-reverse | TTTCTGGCCACGTCCAGTTT |
| *VCAM1*-forward | GGACCACATCTACGCTGACAA |
| *VCAM1*-reverse | CTCCAGAGGGCCACTCAAAT |
| *SDC2*-forward | TCGGCGGAGTCGAGAGC |
| *SDC2*-reverse | AGTGGTCGAGATGTTGTCAGC |
| *CDH2*-forward | TGGGAAATGGAAACTTGATGGC |
| *CDH2*-reverse | AATCTGCAGGCTCACTGCTC |
| *GAPDH*-forward | TCCAAAAATCAAGTGGGGCGA |
| *GAPDH*-reverse | TGATGACCCTTTTGGCTCCC |
