# Supplementary material for: GNG5 is a novel oncogene associated with cell migration, proliferation, and poor prognosis in glioma
Source: Cancer Cell Int. 2021 Jun 7;21:297. doi: 10.1186/s12935-021-01935-7 (PMC8186147; doi:10.1186/s12935-021-01935-7)
Supplement: Supplementary file 3 — Additional file 3: Table S3. Clinical characteristics of glioma patients based on CGGA RNA-seq data. [file 12935_2021_1935_MOESM3_ESM.docx]

| Clinical characteristics | | No. of patients | Percentage(%) |
| --- | --- | --- | --- |
| PRS_type | Primary | 502 | 49.31 |
|  | Recurrent | 222 | 21.81 |
|  | Secondary | 25 | 2.46 |
|  | Missing | 269 | 26.42 |
| Histology | A | 55 | 5.40 |
|  | AA | 39 | 3.83 |
|  | AO | 22 | 2.16 |
|  | AOA | 80 | 7.86 |
|  | GBM | 176 | 17.29 |
|  | O | 35 | 3.44 |
|  | OA | 95 | 9.33 |
|  | rA | 20 | 1.96 |
|  | rAA | 36 | 3.54 |
|  | rAO | 15 | 1.47 |
|  | rAOA | 48 | 4.72 |
|  | rGBM | 90 | 8.84 |
|  | rO | 4 | 0.39 |
|  | rOA | 9 | 0.88 |
|  | sGBM | 25 | 2.46 |
|  | Missing | 269 | 26.42 |
| Grade | WHO II | 218 | 21.41 |
|  | WHO III | 240 | 23.58 |
|  | WHO IV | 291 | 28.59 |
|  | Missing | 269 | 26.42 |
| Gender | Male | 442 | 43.42 |
|  | Female | 307 | 30.16 |
|  | Missing | 269 | 26.42 |
| Age | average_age | 43.256 |  |
|  | (8-79) |  |  |
| OS | average_year | 3.223 |  |
|  | (0-11.984)years |  |  |
|  |  |  |  |
| Radio_status | Yes | 625 | 61.39 |
|  | No | 124 | 12.18 |
|  | Missing | 269 | 26.42 |
| Chemo_status | Yes | 520 | 51.08 |
|  | No | 229 | 22.50 |
|  | Missing | 269 | 26.42 |
| IDH_mutation_status | Mutant | 410 | 40.28 |
|  | Wildtype | 339 | 33.30 |
|  | Missing | 269 | 26.42 |
| 1p19q_codeletion_status | Codel | 155 | 15.23 |
|  | Non-codel | 594 | 58.35 |
|  | Missing | 269 | 26.42 |

A: astrocytomas; AA: anaplastic astrocytomas; AO: anaplastic oligodendrogliomas; AOA: anaplastic oligoastrocytomas; GBM: glioblastoma; IDH: isocitrate dehydrogenase; O: oligodendrogliomas; OA: oligoastrocytomas; OS: overall survival; PRS: primary recurrence or secondary; rA: recurrent astrocytomas; rAA: recurrent anaplastic astrocytomas; rAO: recurrent anaplastic oligodendrogliomas; rAOA: recurrent anaplastic oligoastrocytomas; rGBM: recurrent glioblastoma; rO: recurrent oligodendrogliomas; rOA: recurrent oligoastrocytomas; sGBM: secondary glioblastoma.
