# Supplementary figures and images for: GNG5 is a novel oncogene associated with cell migration, proliferation, and poor prognosis in glioma
Source: Cancer Cell Int. 2021 Jun 7;21:297. doi: 10.1186/s12935-021-01935-7 (PMC8186147; doi:10.1186/s12935-021-01935-7)

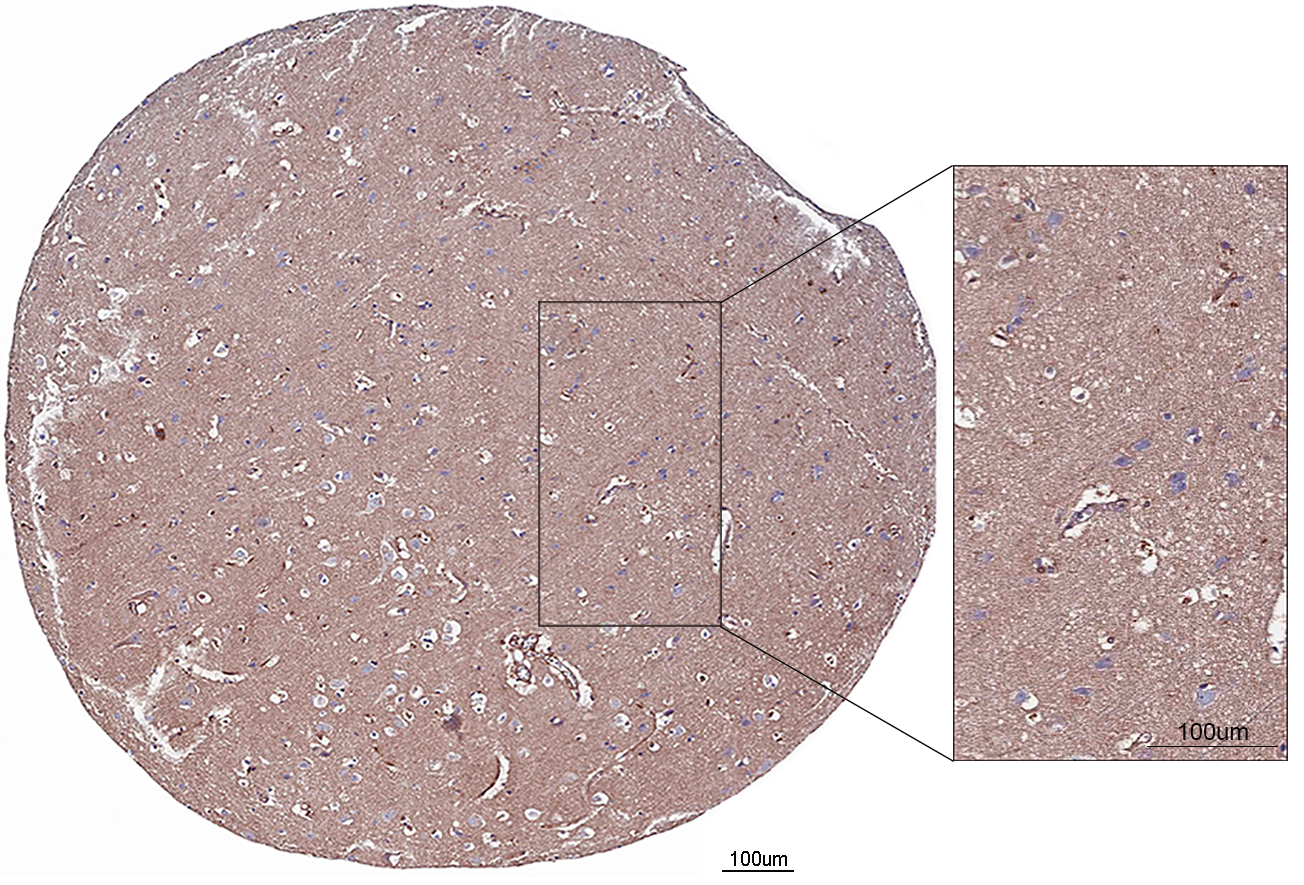

Supplement: Supplementary file 4 — Additional file 4: Figure S1: Correlation between GNG5 expression level and overall survival of glioma patients. (A): Immunohistochemical results of GNG5 in glioma and normal tissues based on human protein atlas. LGG: low grade glioma, HGG: high grade glioma; (B): Kaplan-Meier curve based on TCGA database; (C): Differences in GNG5 expression in patients with different survival periods based on GEO (GSE 53733). [file 12935_2021_1935_MOESM4_ESM.zip › Figure S1-A1.tif]

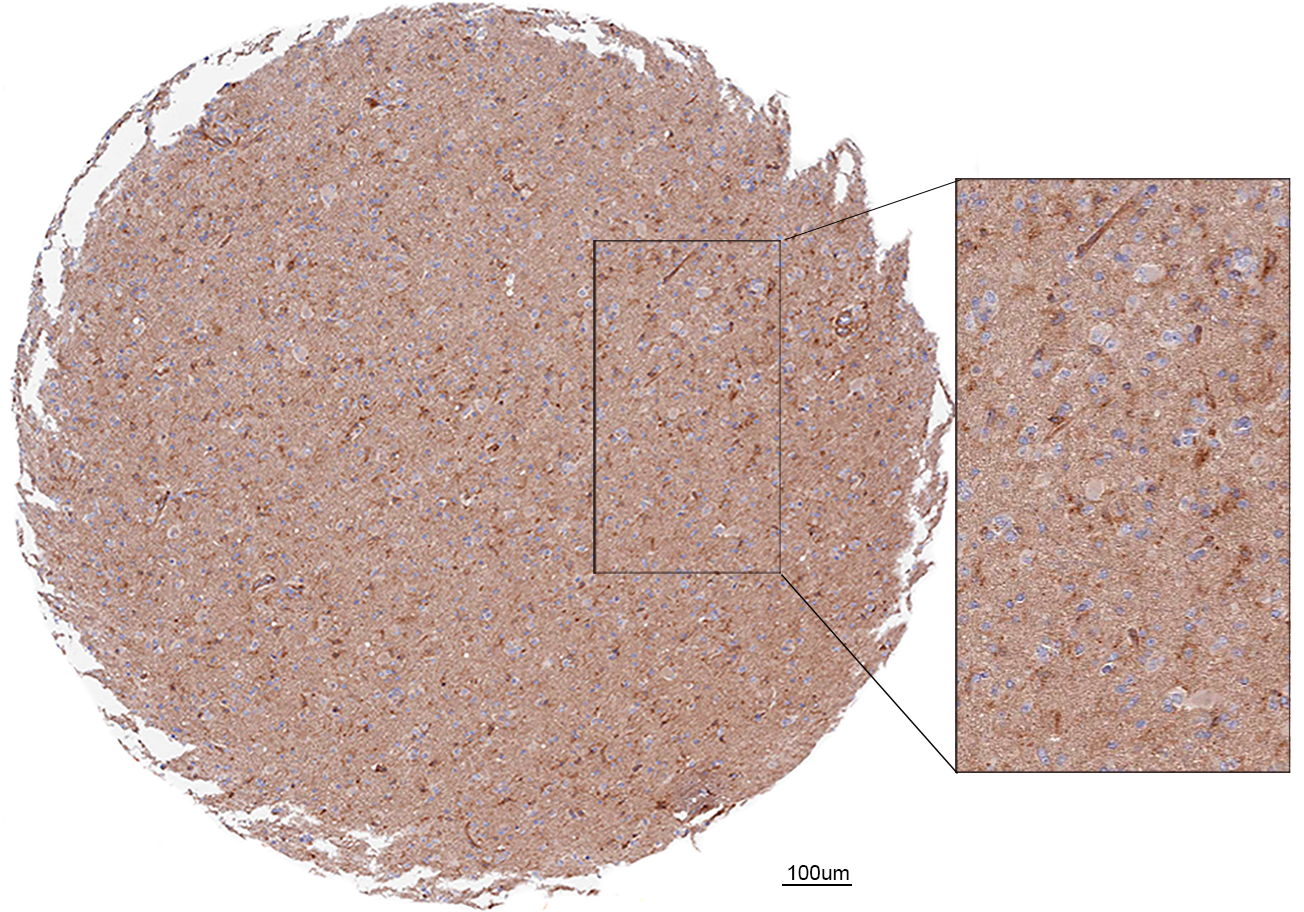

Supplement: Supplementary file 4 — Additional file 4: Figure S1: Correlation between GNG5 expression level and overall survival of glioma patients. (A): Immunohistochemical results of GNG5 in glioma and normal tissues based on human protein atlas. LGG: low grade glioma, HGG: high grade glioma; (B): Kaplan-Meier curve based on TCGA database; (C): Differences in GNG5 expression in patients with different survival periods based on GEO (GSE 53733). [file 12935_2021_1935_MOESM4_ESM.zip › Figure S1-A2.tif]

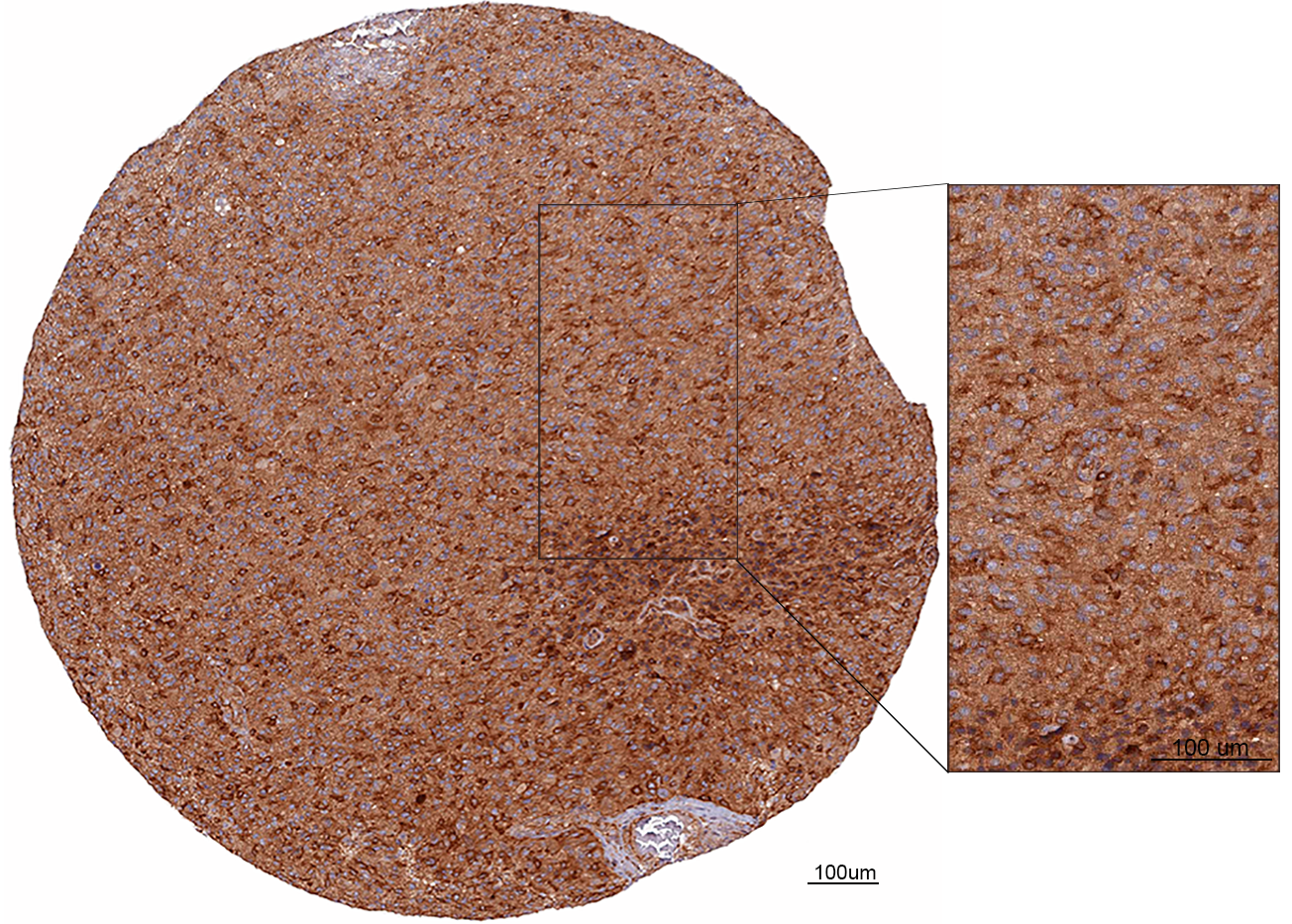

Supplement: Supplementary file 4 — Additional file 4: Figure S1: Correlation between GNG5 expression level and overall survival of glioma patients. (A): Immunohistochemical results of GNG5 in glioma and normal tissues based on human protein atlas. LGG: low grade glioma, HGG: high grade glioma; (B): Kaplan-Meier curve based on TCGA database; (C): Differences in GNG5 expression in patients with different survival periods based on GEO (GSE 53733). [file 12935_2021_1935_MOESM4_ESM.zip › Figure S1-A3.tif]

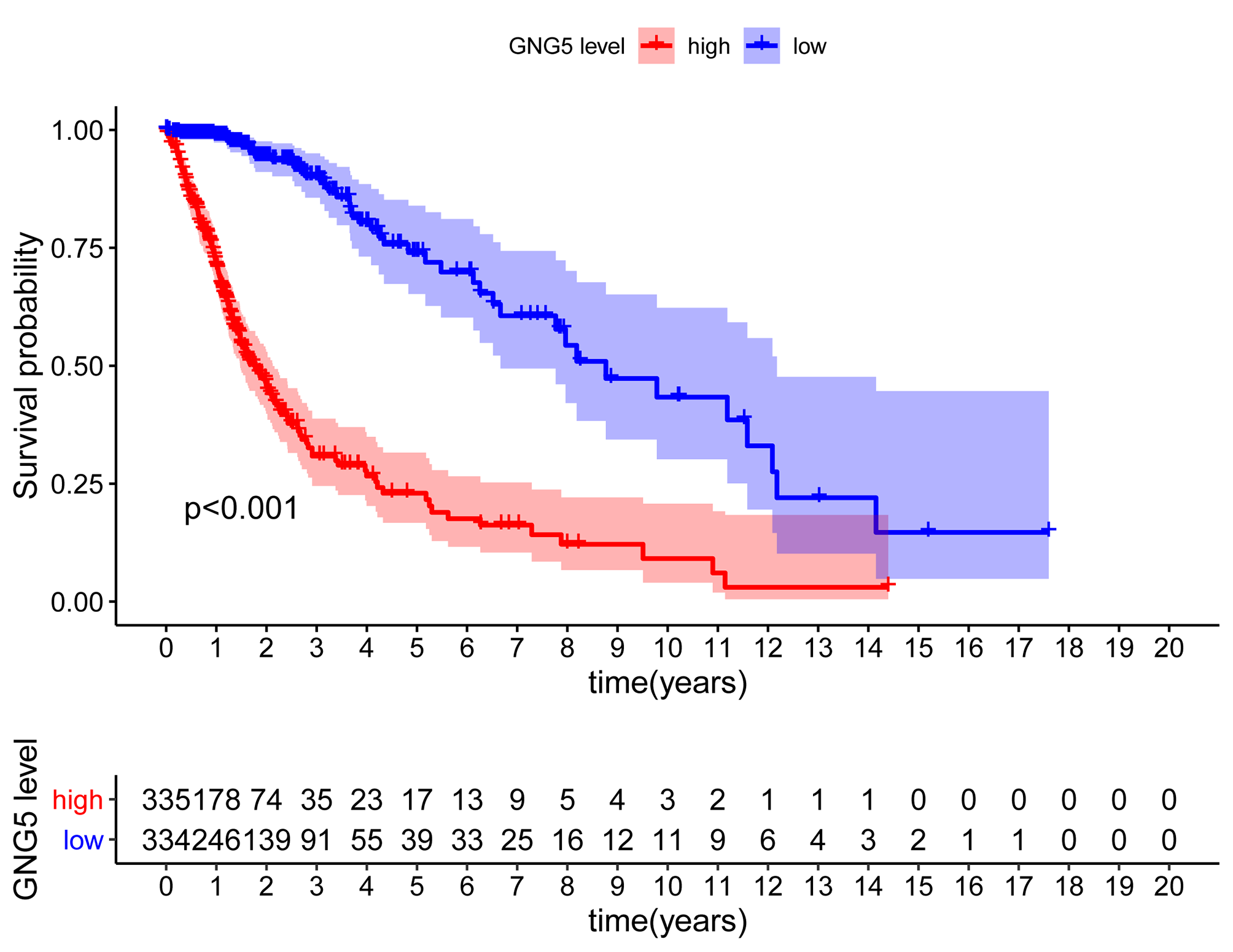

Supplement: Supplementary file 4 — Additional file 4: Figure S1: Correlation between GNG5 expression level and overall survival of glioma patients. (A): Immunohistochemical results of GNG5 in glioma and normal tissues based on human protein atlas. LGG: low grade glioma, HGG: high grade glioma; (B): Kaplan-Meier curve based on TCGA database; (C): Differences in GNG5 expression in patients with different survival periods based on GEO (GSE 53733). [file 12935_2021_1935_MOESM4_ESM.zip › Figure S1B.tif]

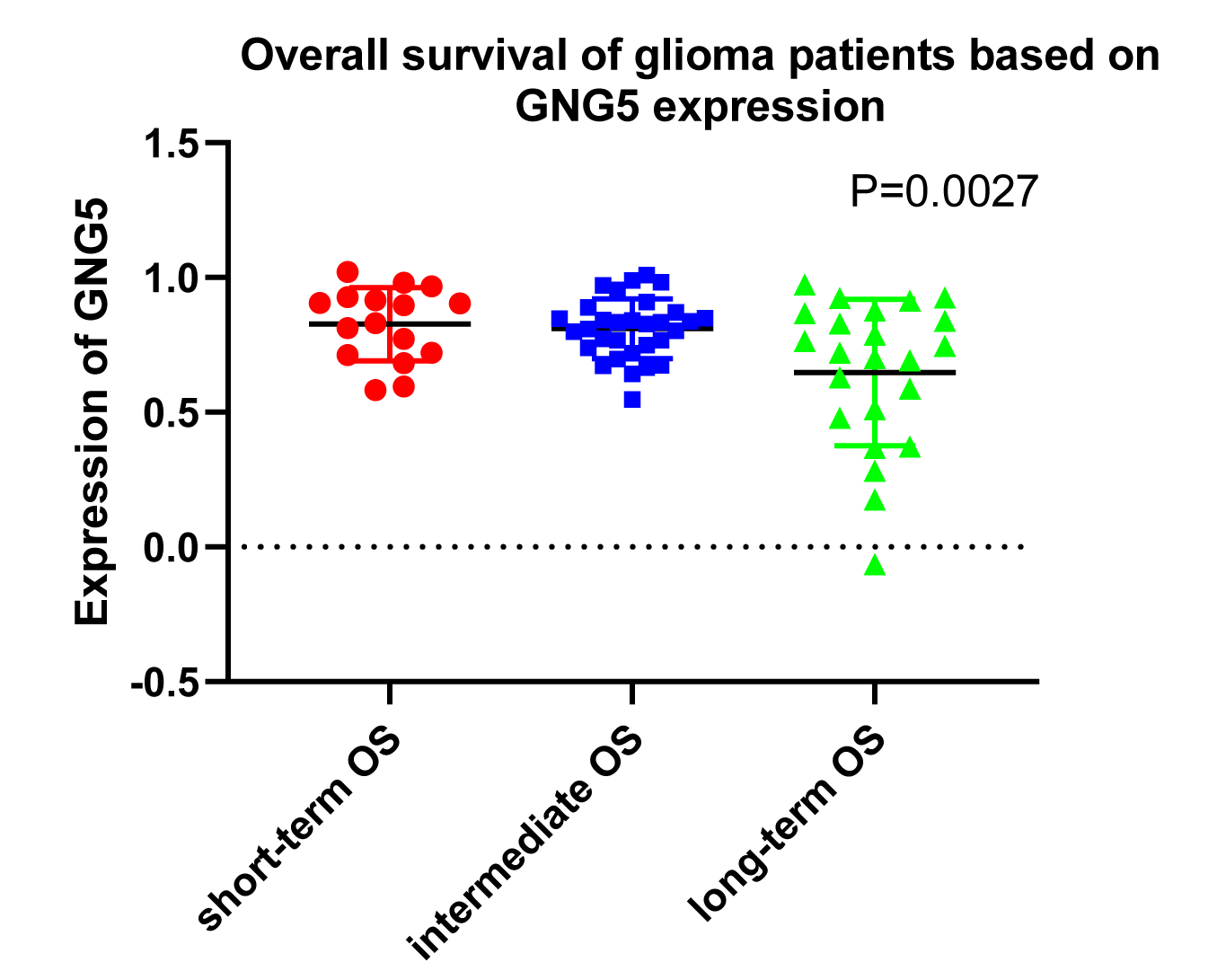

Supplement: Supplementary file 4 — Additional file 4: Figure S1: Correlation between GNG5 expression level and overall survival of glioma patients. (A): Immunohistochemical results of GNG5 in glioma and normal tissues based on human protein atlas. LGG: low grade glioma, HGG: high grade glioma; (B): Kaplan-Meier curve based on TCGA database; (C): Differences in GNG5 expression in patients with different survival periods based on GEO (GSE 53733). [file 12935_2021_1935_MOESM4_ESM.zip › Figure S1C.tif]

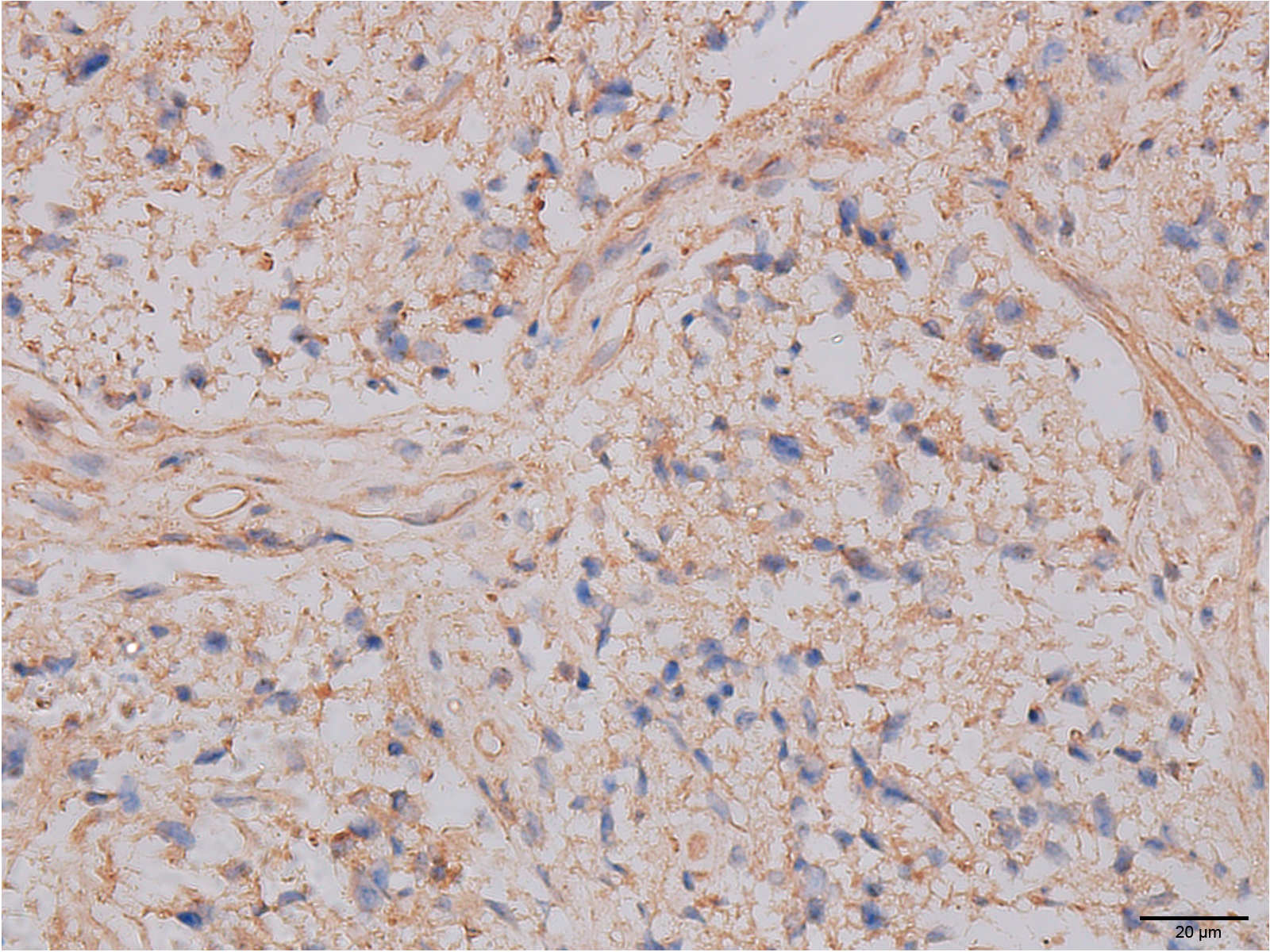

Supplement: Supplementary file 5 — Additional file 5: Figure S2: Correlation between IDH1 R132H and GNG5 in glioma samples. (A): Representative results of IDH1 R132H immunohistochemical staining in GNG5 positive tissue; (B): correlation analysis of GNG5 and IDH1 R132H based on the clinical sample. *P = 0.0309. [file 12935_2021_1935_MOESM5_ESM.zip › Figure S2_A1_tissue2_GNG5.tif]

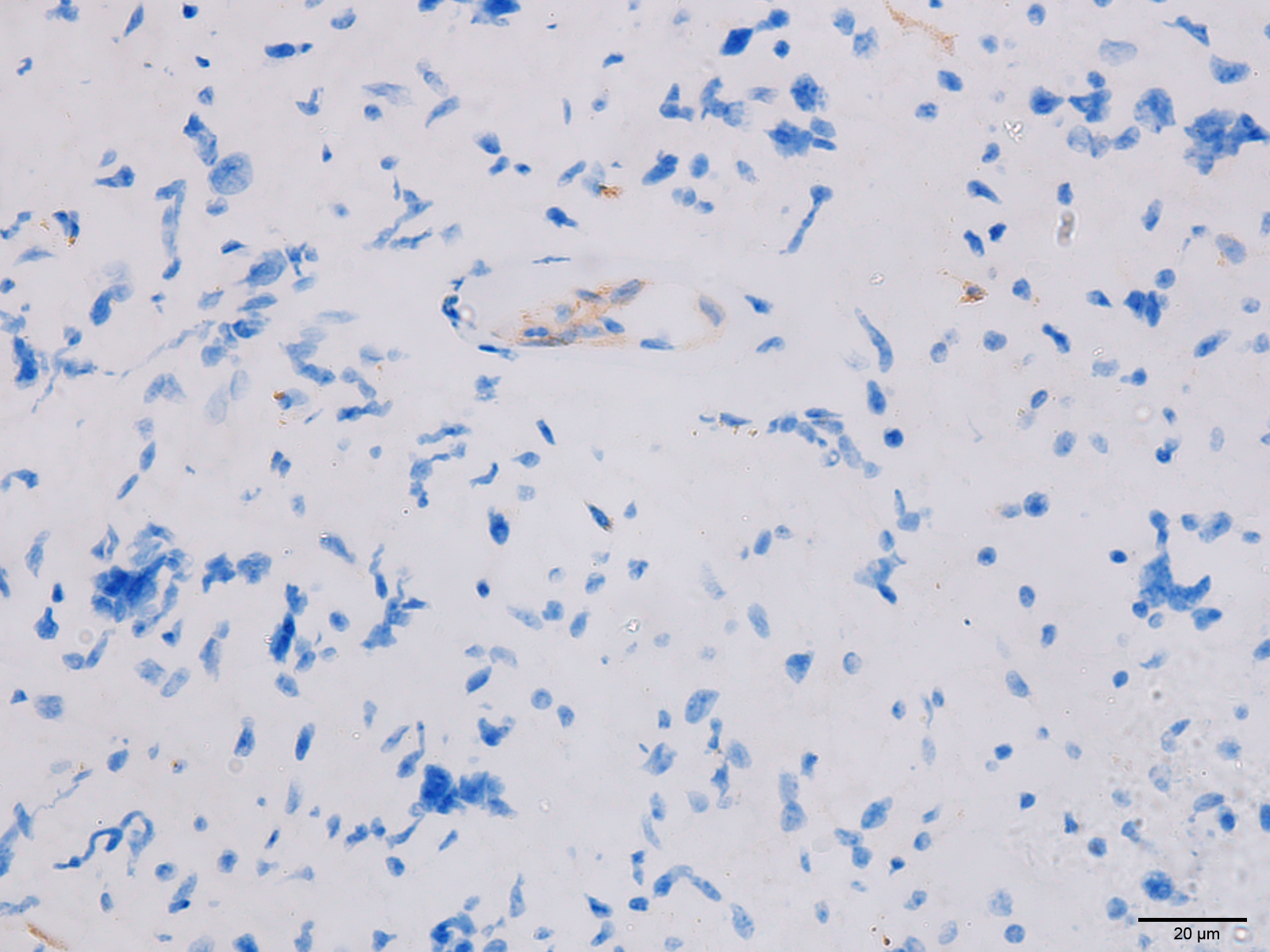

Supplement: Supplementary file 5 — Additional file 5: Figure S2: Correlation between IDH1 R132H and GNG5 in glioma samples. (A): Representative results of IDH1 R132H immunohistochemical staining in GNG5 positive tissue; (B): correlation analysis of GNG5 and IDH1 R132H based on the clinical sample. *P = 0.0309. [file 12935_2021_1935_MOESM5_ESM.zip › Figure S2_A2_tissue2_IDH1.tif]

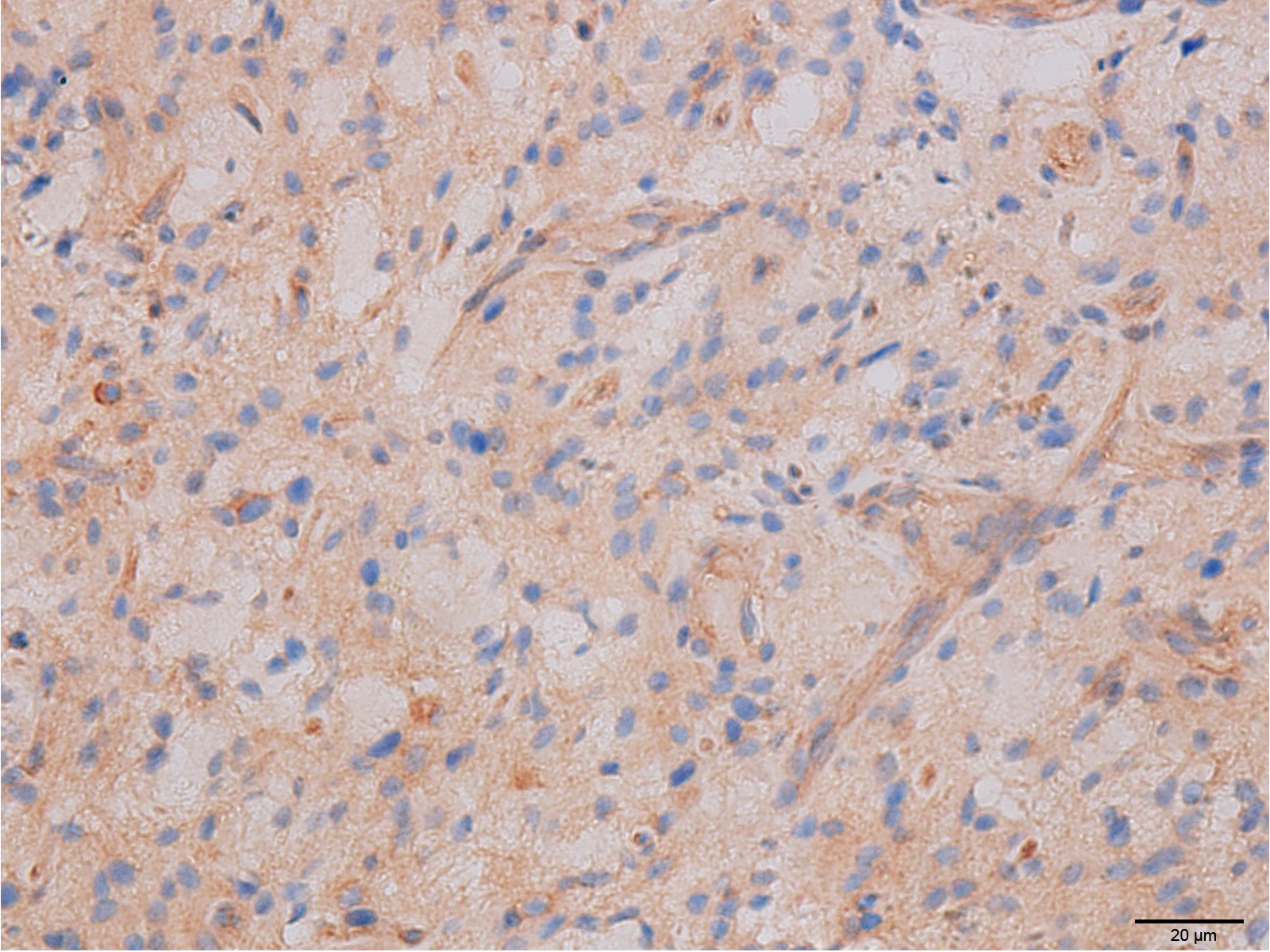

Supplement: Supplementary file 5 — Additional file 5: Figure S2: Correlation between IDH1 R132H and GNG5 in glioma samples. (A): Representative results of IDH1 R132H immunohistochemical staining in GNG5 positive tissue; (B): correlation analysis of GNG5 and IDH1 R132H based on the clinical sample. *P = 0.0309. [file 12935_2021_1935_MOESM5_ESM.zip › Figure S2_A3_tissue4_GNG5.tif]

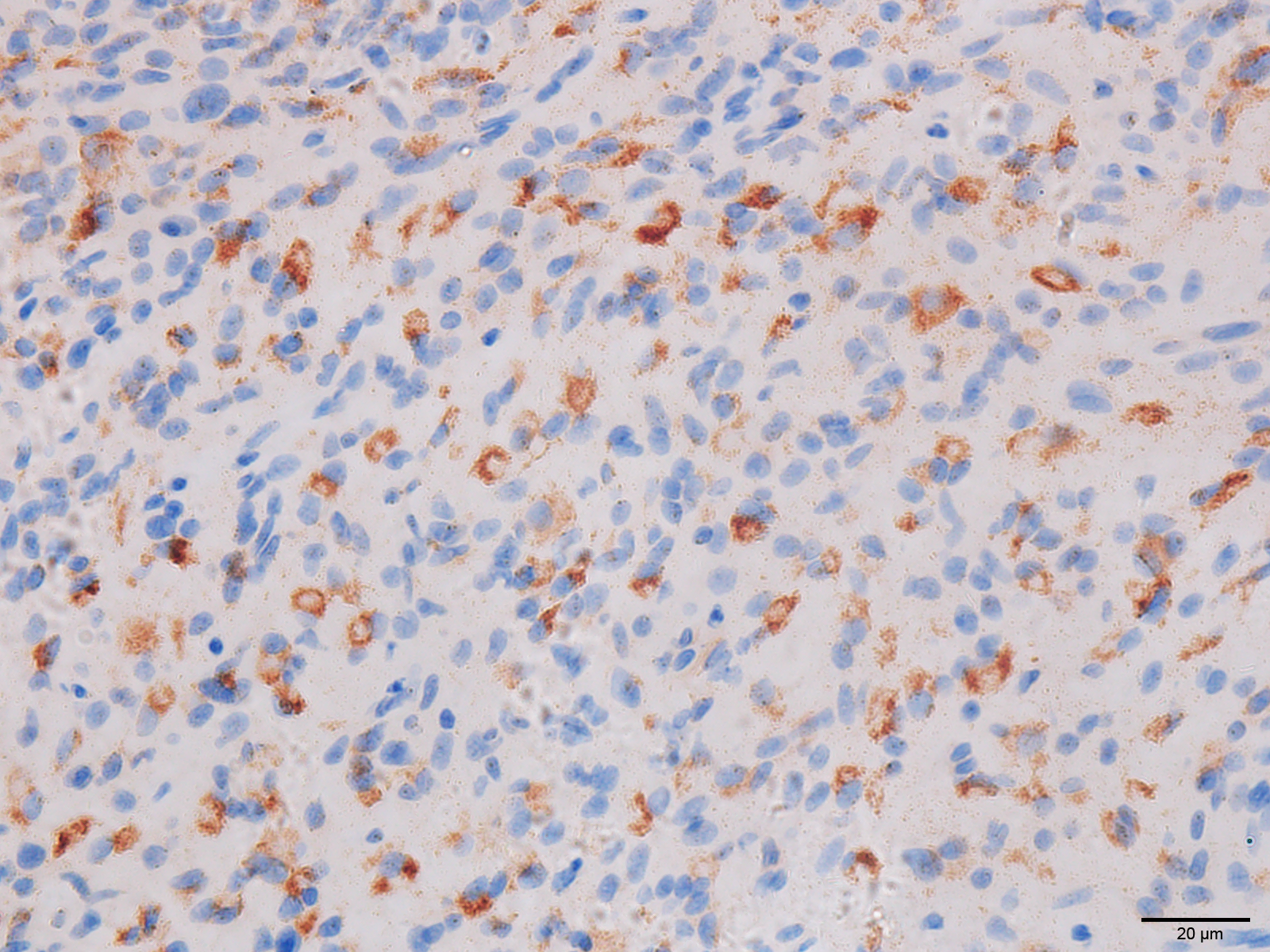

Supplement: Supplementary file 5 — Additional file 5: Figure S2: Correlation between IDH1 R132H and GNG5 in glioma samples. (A): Representative results of IDH1 R132H immunohistochemical staining in GNG5 positive tissue; (B): correlation analysis of GNG5 and IDH1 R132H based on the clinical sample. *P = 0.0309. [file 12935_2021_1935_MOESM5_ESM.zip › Figure S2_A4_tissue4_IDH1.tif]

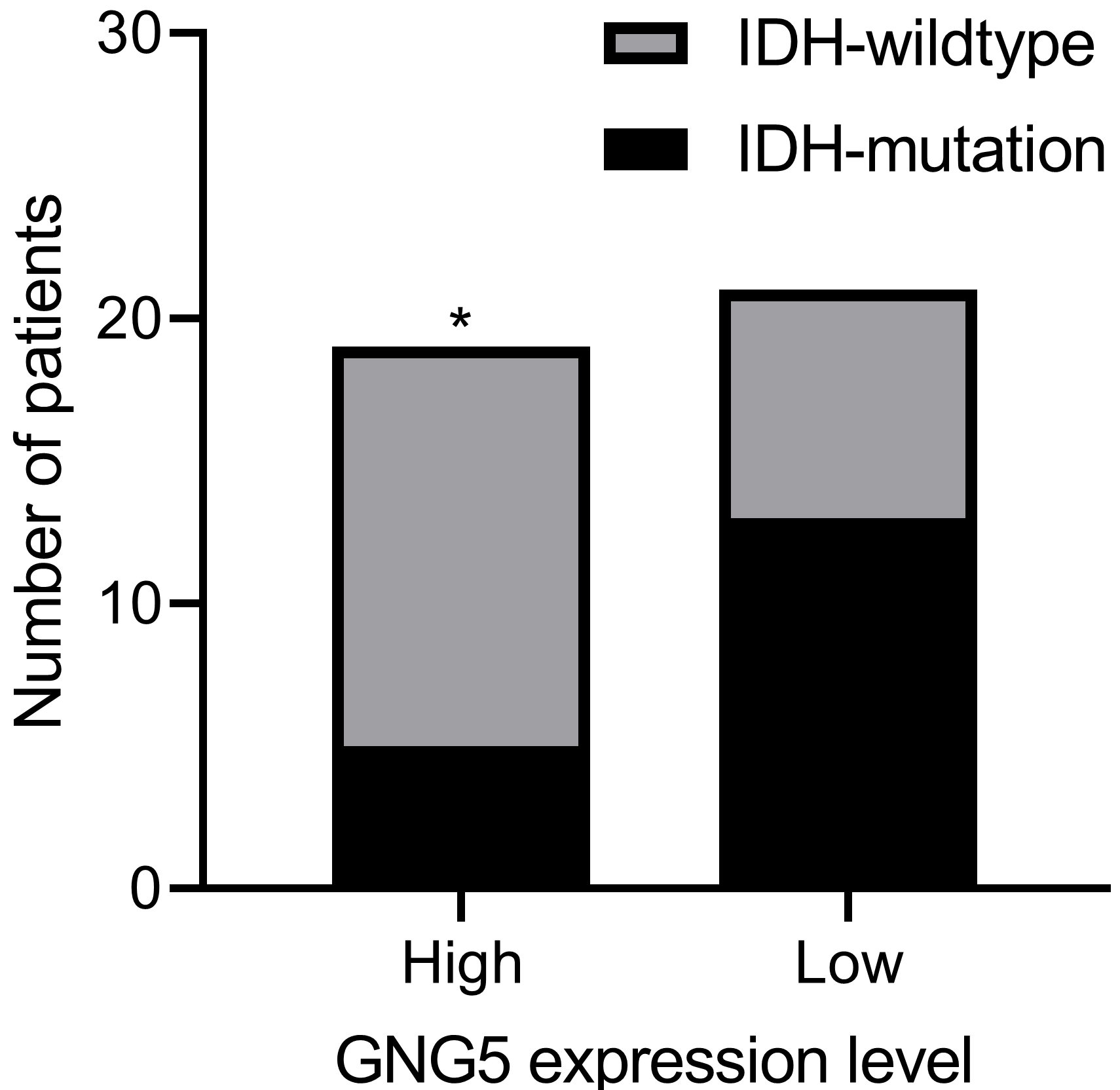

Supplement: Supplementary file 5 — Additional file 5: Figure S2: Correlation between IDH1 R132H and GNG5 in glioma samples. (A): Representative results of IDH1 R132H immunohistochemical staining in GNG5 positive tissue; (B): correlation analysis of GNG5 and IDH1 R132H based on the clinical sample. *P = 0.0309. [file 12935_2021_1935_MOESM5_ESM.zip › Figure S2B.tif]

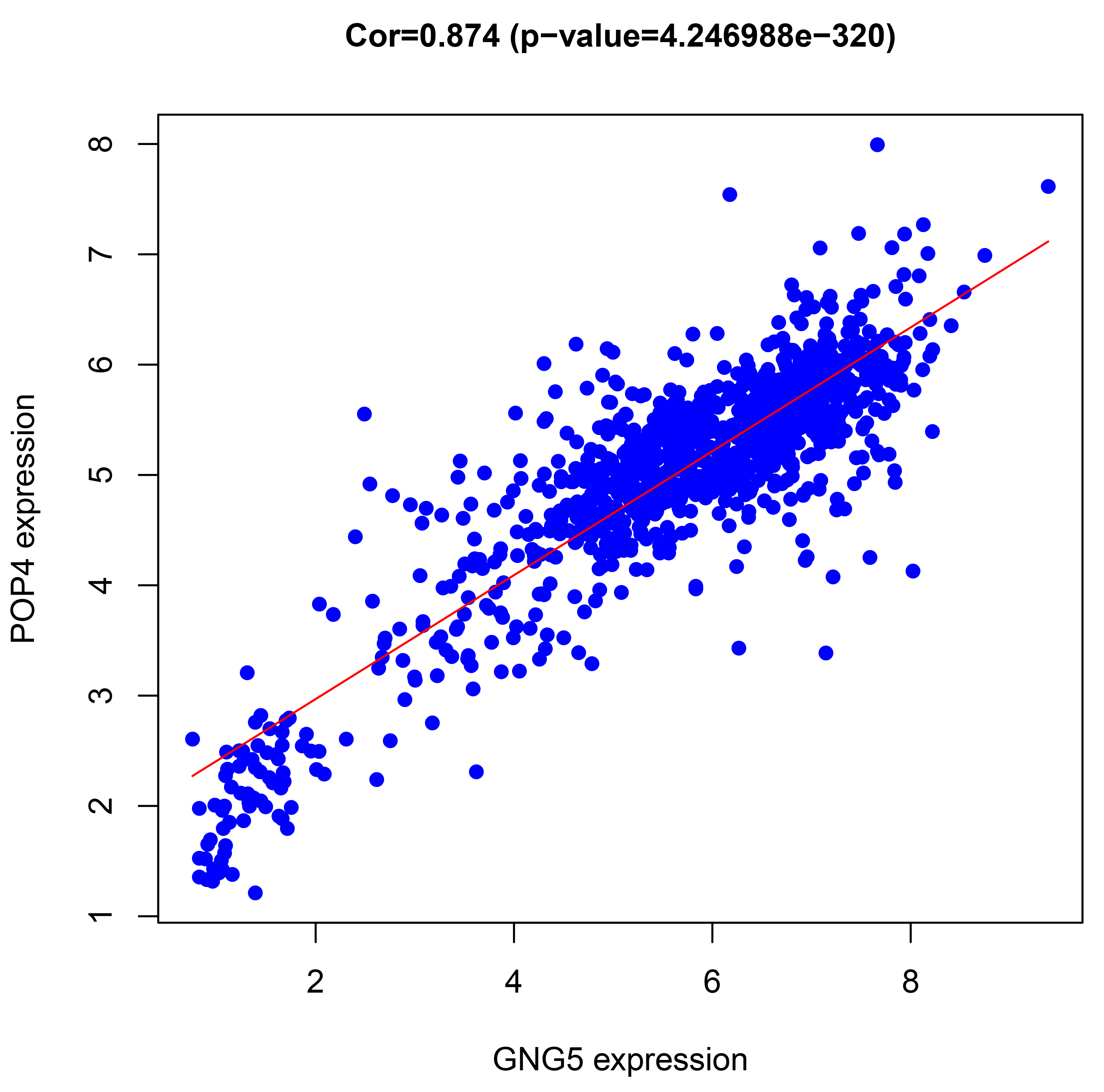

Supplement: Supplementary file 6 — Additional file 6: Figure S3: Co-expression analysis of GNG5 and GSEA enrichment analysis results. The correlation between GNG5 and POP4 (A), RER1 (B), ATRNL1 (C), TUB (D); GSEA enrichment analysis of the ECM-receptor interaction (E), the focal adhesion (F), the toll-like receptor signaling pathway (G) and the nod-like receptor signaling pathway (H). [file 12935_2021_1935_MOESM6_ESM.zip › Figure S3A.tif]

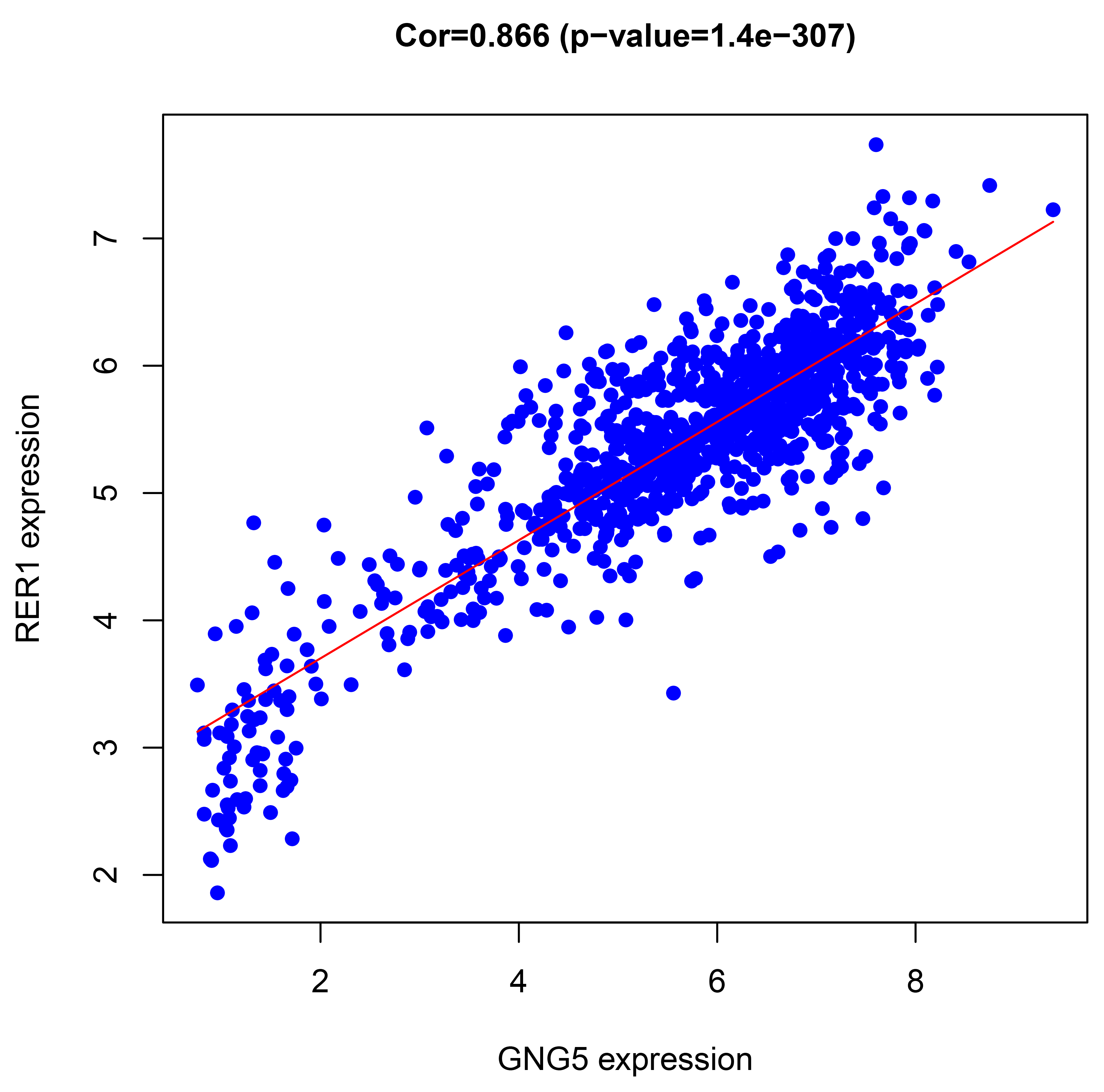

Supplement: Supplementary file 6 — Additional file 6: Figure S3: Co-expression analysis of GNG5 and GSEA enrichment analysis results. The correlation between GNG5 and POP4 (A), RER1 (B), ATRNL1 (C), TUB (D); GSEA enrichment analysis of the ECM-receptor interaction (E), the focal adhesion (F), the toll-like receptor signaling pathway (G) and the nod-like receptor signaling pathway (H). [file 12935_2021_1935_MOESM6_ESM.zip › Figure S3B.tif]

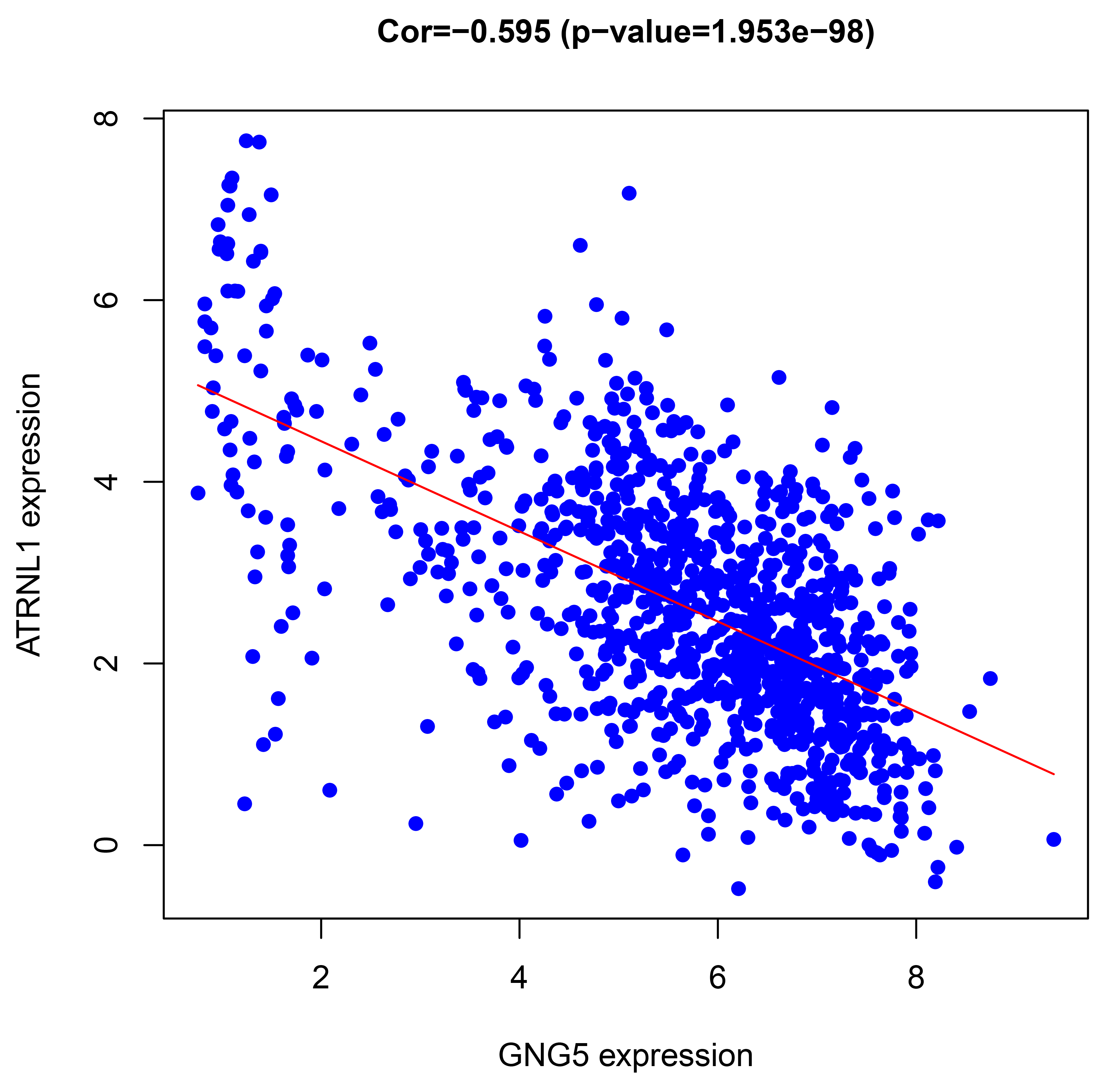

Supplement: Supplementary file 6 — Additional file 6: Figure S3: Co-expression analysis of GNG5 and GSEA enrichment analysis results. The correlation between GNG5 and POP4 (A), RER1 (B), ATRNL1 (C), TUB (D); GSEA enrichment analysis of the ECM-receptor interaction (E), the focal adhesion (F), the toll-like receptor signaling pathway (G) and the nod-like receptor signaling pathway (H). [file 12935_2021_1935_MOESM6_ESM.zip › Figure S3C.tif]

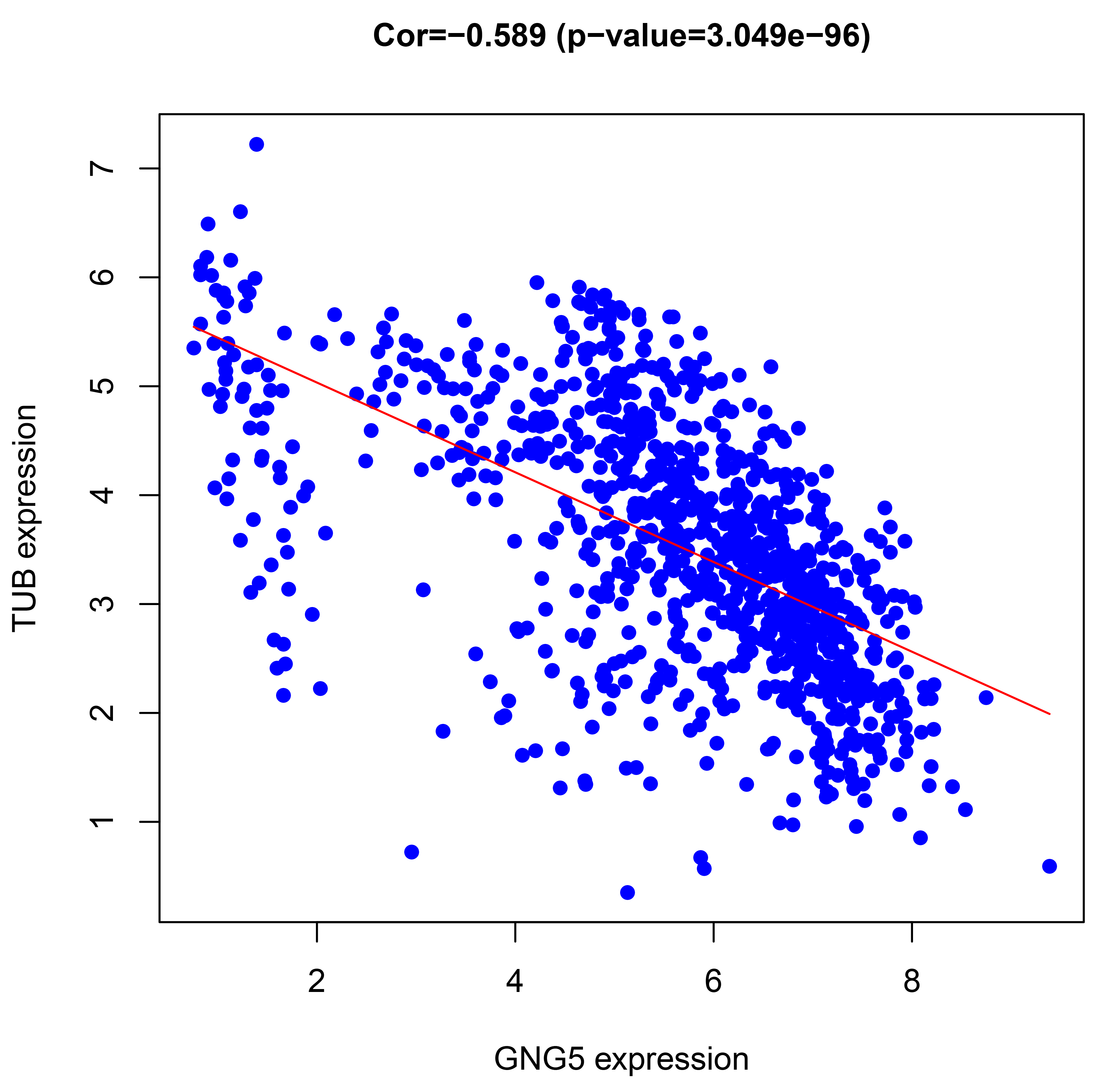

Supplement: Supplementary file 6 — Additional file 6: Figure S3: Co-expression analysis of GNG5 and GSEA enrichment analysis results. The correlation between GNG5 and POP4 (A), RER1 (B), ATRNL1 (C), TUB (D); GSEA enrichment analysis of the ECM-receptor interaction (E), the focal adhesion (F), the toll-like receptor signaling pathway (G) and the nod-like receptor signaling pathway (H). [file 12935_2021_1935_MOESM6_ESM.zip › Figure S3D.tif]

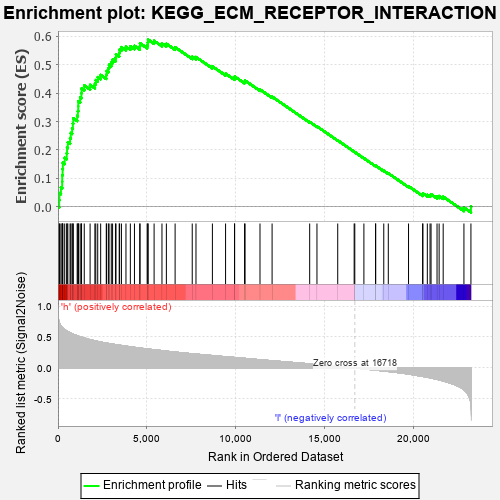

Supplement: Supplementary file 6 — Additional file 6: Figure S3: Co-expression analysis of GNG5 and GSEA enrichment analysis results. The correlation between GNG5 and POP4 (A), RER1 (B), ATRNL1 (C), TUB (D); GSEA enrichment analysis of the ECM-receptor interaction (E), the focal adhesion (F), the toll-like receptor signaling pathway (G) and the nod-like receptor signaling pathway (H). [file 12935_2021_1935_MOESM6_ESM.zip › Figure S3E.tif]

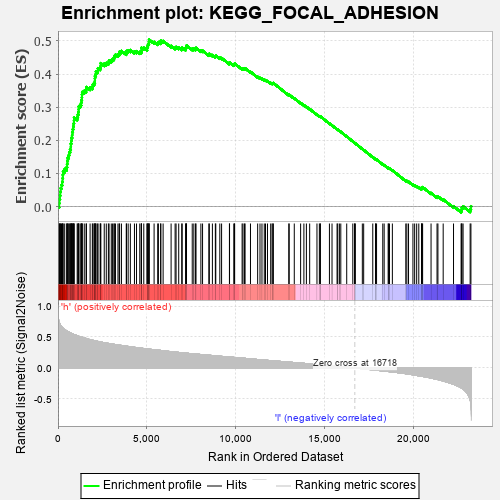

Supplement: Supplementary file 6 — Additional file 6: Figure S3: Co-expression analysis of GNG5 and GSEA enrichment analysis results. The correlation between GNG5 and POP4 (A), RER1 (B), ATRNL1 (C), TUB (D); GSEA enrichment analysis of the ECM-receptor interaction (E), the focal adhesion (F), the toll-like receptor signaling pathway (G) and the nod-like receptor signaling pathway (H). [file 12935_2021_1935_MOESM6_ESM.zip › Figure S3F.tif]

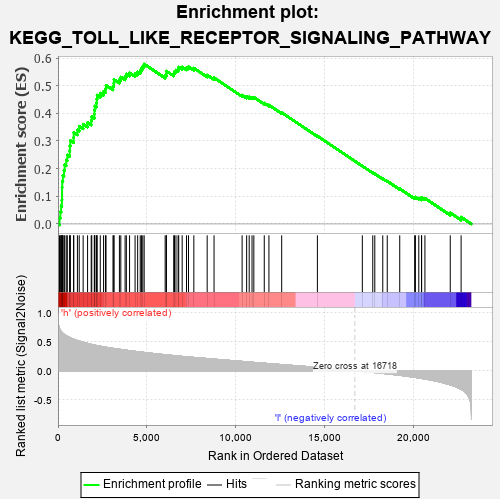

Supplement: Supplementary file 6 — Additional file 6: Figure S3: Co-expression analysis of GNG5 and GSEA enrichment analysis results. The correlation between GNG5 and POP4 (A), RER1 (B), ATRNL1 (C), TUB (D); GSEA enrichment analysis of the ECM-receptor interaction (E), the focal adhesion (F), the toll-like receptor signaling pathway (G) and the nod-like receptor signaling pathway (H). [file 12935_2021_1935_MOESM6_ESM.zip › Figure S3G.tif]

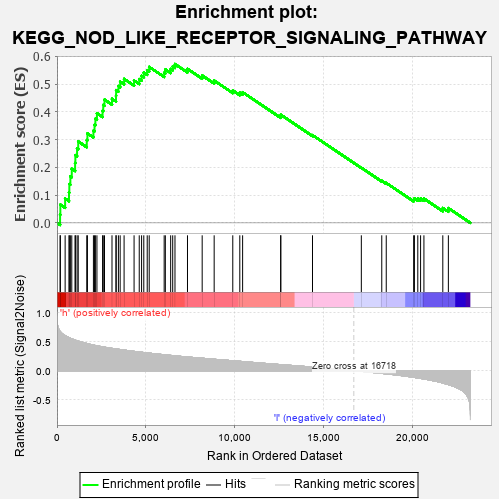

Supplement: Supplementary file 6 — Additional file 6: Figure S3: Co-expression analysis of GNG5 and GSEA enrichment analysis results. The correlation between GNG5 and POP4 (A), RER1 (B), ATRNL1 (C), TUB (D); GSEA enrichment analysis of the ECM-receptor interaction (E), the focal adhesion (F), the toll-like receptor signaling pathway (G) and the nod-like receptor signaling pathway (H). [file 12935_2021_1935_MOESM6_ESM.zip › Figure S3H.tif]
